# Hidden white and black feather layers enhance plumage coloration in tanagers and other songbirds

**DOI:** 10.1126/sciadv.adw5857

**Published:** 2025-07-23

**Authors:** Rosalyn M. Price-Waldman, Jarome R. Ali, Allison J. Shultz, Benedict G. Hogan, Mary Caswell Stoddard

**Affiliations:** ^1^ Department of Ecology and Evolutionary Biology, Princeton University, Princeton, NJ 08544, USA.; ^2^ Department of Anthropology, New York University, New York, NY 10003, USA.; ^3^ Ornithology Department, Natural History Museum of Los Angeles County, Los Angeles, CA 90007, USA.

## Abstract

Birds are renowned for their diverse and colorful plumage. Here, we demonstrate that vibrant plumage in the tanager genus *Tangara* is substantially intensified by a “hidden” layer of achromatic (white or black) plumage concealed beneath the outermost colorful layer. Using hyperspectral imaging, multispectral photography, microspectrophotometry, reflectance spectrophotometry, and optical modeling, we show that hidden white and black feather layers are systematically distributed on the body to enhance the brightness and saturation of carotenoid-pigmented and structurally colored plumage, respectively, by increasing or decreasing the amount of backscattered light that interacts with pigments or nanostructures. We compare male and female coloration and show that sexual dichromatism in some *Tangara* carotenoid plumage stems primarily from white layers in males and black layers in females rather than from differences in carotenoid pigmentation. Last, we find that white and black hidden feather layers are widespread in colorful passerines. Hidden feather layers likely play a previously overlooked but critical role in colorful plumage evolution in birds.

## Teaser

Colorful songbirds use hidden white or black feather layers to enhance plumage color, an optical trick well known to artists.

## INTRODUCTION

Animal integuments (skin, scales, feathers, and fur) are often specialized to produce dazzling optical effects, from transparency and translucency (*1*–*3*) to deeply absorbent ultra- and superblack colors (*4*–*6*) to rainbow iridescence (*7*–*9*). Characterizing the physical bases of these diverse optical effects in animal tissues is a priority for animal color research (*10*, *11*) and has widespread applications, from revealing the function and evolution of ornamentation to inspiring technological and medical advances (*12*, *13*).

Feathers are among the most colorful animal integuments, and avian plumage color has been a classic system for the study of natural and sexual selection (*14*–*16*). Feathers are keratinous structures made up of hierarchically nested branches. The main shaft (the rachis) contains paired branches called barbs, which in turn contain a central shaft (the barb ramus) and smaller branches called barbules (*17*). Colors are produced by pigments or nanostructures in the rachis, the barb ramus, or barbules. Among extant birds, melanin pigments—which give rise to black, brown, and rusty red colors—are ancestral and widespread. Over evolutionary time, many bird lineages have evolved new pigments and/or structural colors that expand the avian plumage palette (*18*). Most red, yellow, and orange colors are produced by diet-derived carotenoid pigments embedded in feather keratin (*19*). Most ultraviolet, violet, and blue colors are produced by coherent scattering of light from nanostructures made of keratin and air within the rami of the feather barbs (noniridescent structural colors) or by ordered arrays of melanosomes, keratin, and air within feather barbules (iridescent structural colors) (*20*, *21*). Both carotenoid pigmentation and structural coloration have evolved many times across birds (*18*, *21*, *22*), and pigments and structural colors are often combined within the feather to further expand the achievable range of colors (*18*, *23*).

Bird plumages comprise many types of feathers, including contour (body) feathers (*17*, *24*). Birds typically have thousands of contour feathers (*25*), which are attached to the skin and positioned on the body like shingles on a roof. Contour feathers have a pennaceous region (the tip) and a plumulaceous region (the base) (*24*, *26*). In contour feathers, the carotenoid pigments and nanostructures that produce colors are generally restricted to the pennaceous tips, while the plumulaceous bases are generally white, gray, or brown as a result of sparsely distributed melanin pigments (*24*). The results are an outermost colorful layer of plumage formed by the pennaceous feather tips and an innermost layer of downy plumage formed by the plumulaceous bases. Studies on plumage coloration generally assume that the optical properties of the outermost pennaceous layer determine plumage color and that only this layer is subject to selection for signaling. However, the “hidden” regions of feathers also reflect and absorb light, and the pennaceous feather layer is not opaque—raising the possibility that there may be critical but overlooked optical interactions among the visible and hidden feather layers. What are the properties of the hidden parts of contour feathers, and does light reflection or absorption by these hidden feather layers have optical consequences for plumage coloration?

**Fig. 1.**
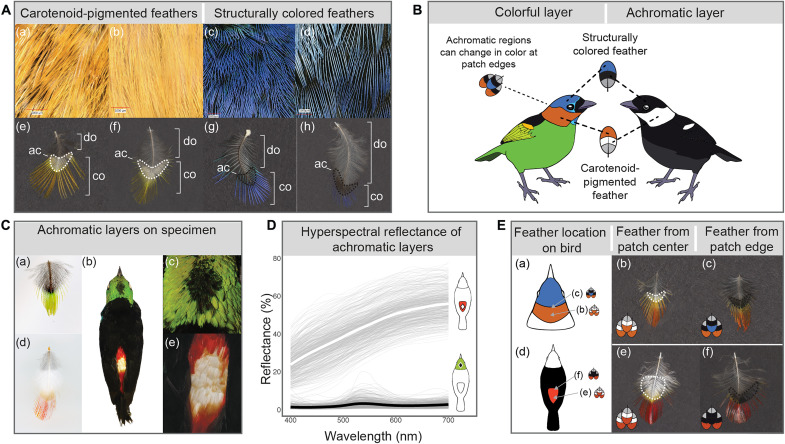
Feather achromatic regions and hidden achromatic layers. (**A**) Confocal microscope images of feathers layered on specimens [(a) to (d)] and colorful, achromatic, and downy regions within individual feathers [(e) to (h)]. Colorful, achromatic, and downy feather regions in (e) to (h) are labeled co, ac, and do, respectively, with achromatic regions outlined in white or black. When layered on the body, white and black achromatic regions of individual feathers form a continuous layer of achromatic plumage concealed by the colorful layer of plumage, illustrated in (**B**) and demonstrated on a *T. chilensis* specimen with colorful feather tips removed to expose white and black achromatic layers in (**C**). In (C), (a) and (d) show individual feathers with prominent achromatic regions sampled from a *T. chilensis* crown and rump, respectively, while (b), (c), and (e) show exposed achromatic layers on the specimen. (**D**) Reflectance of *T. chilensis* white and black achromatic layers from (C) measured with hyperspectral imaging (HI). Transparent lines are spectra from 500 random samples from within the white or black achromatic region, and solid lines are the mean reflectance. (**E**) Switch in achromatic region color for carotenoid-pigmented feathers sampled from the centers of red patches (b and e) versus the junctions of those patches with blue structurally colored (c) and black melanin-pigmented (f) patches. The locations of the feather samples are also illustrated (a) and (d). (b) *Tangara cyanocephala* neck (patch center), (c) *T. cyanocephala* neck (edge of patch, adjacent to blue crown), (e) *T. chilensis* rump (patch center), (f) *T. chilensis* rump (edge of patch, adjacent to black back).

**Fig. 2.**
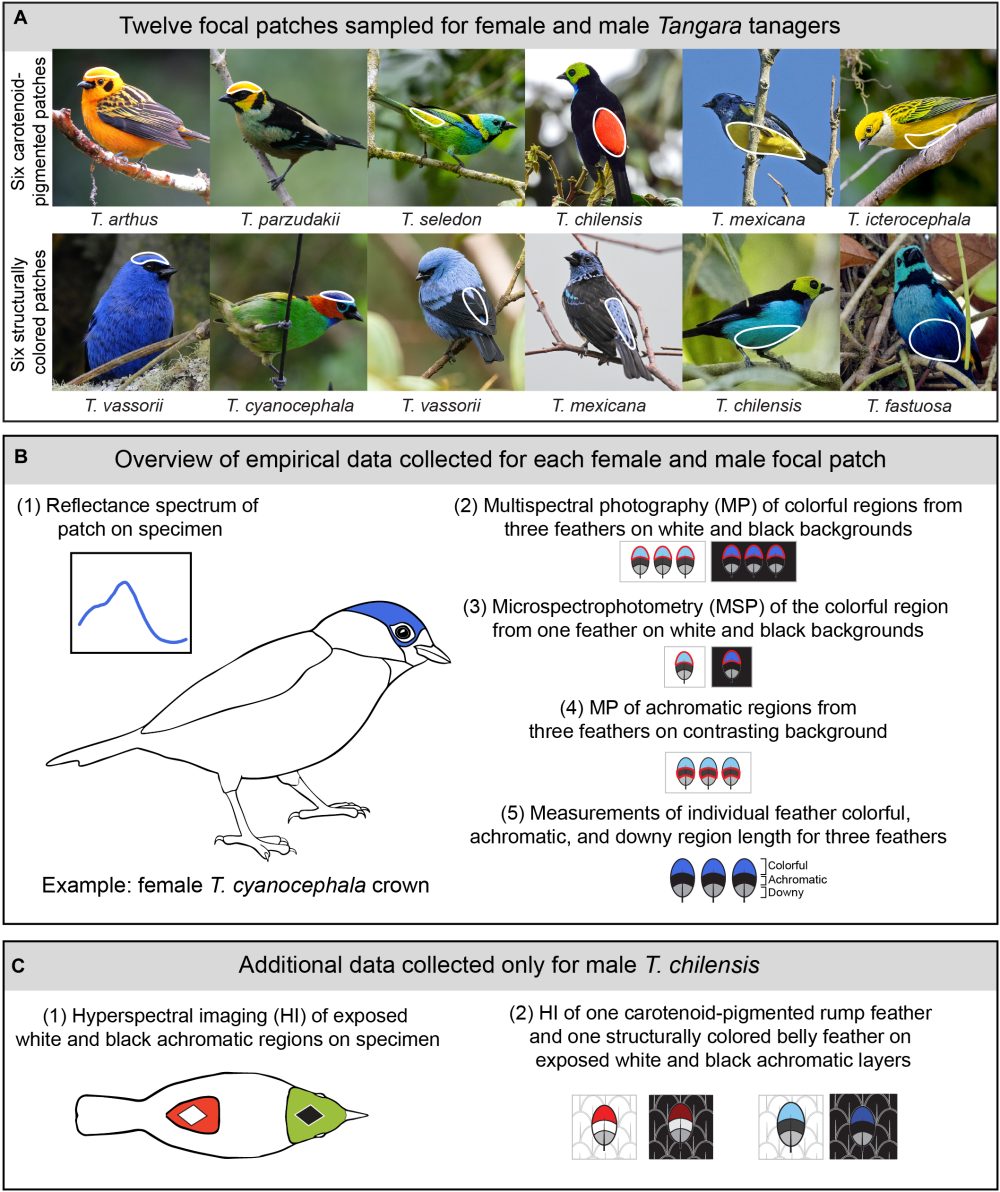
Overview of sampling scheme and data collection. (**A**) We selected six carotenoid-pigmented and six structurally colored patches (crown, rump, and belly patches; outlined in white) to focus on from across the genus *Tangara*. We sampled feathers from females and males for each patch. In (**B**), we provide an overview of the empirical data collected for each of the 12 focal patches for females and males, using the structurally colored crown of a female *T. cyanocephala* as an example. In (**C**), we provide an overview of the additional data we collected only for male *T. chilensis.* Image credits in (A) from top left: Félix Uribe (CC BY 2.0; https://creativecommons.org/licenses/by/2.0/), Ben Keen (CC0; https://creativecommons.org/publicdomain/zero/1.0/deed.en), Thomaz de Carvalho Callado (CC BY 4.0; https://creativecommons.org/licenses/by/4.0/), Ferhat Gundogdu (CC0; https://creativecommons.org/publicdomain/zero/1.0/deed.en), Christoph Moning (CC BY 4.0; https://creativecommons.org/licenses/by/4.0/), Chaz Jackson (CC BY 2.0; https://creativecommons.org/licenses/by/2.0/), Esteban Poveda (CC BY 4.0; https://creativecommons.org/licenses/by/4.0/deed.en), Antoine Guiguet (CC BY 4.0; https://creativecommons.org/licenses/by/4.0/), Chuck Gates (CC BY 2.0; https://creativecommons.org/licenses/by/2.0/legalcode.en), Laura Wolf (CC BY 2.0; https://creativecommons.org/licenses/by/2.0/), Claude Kolwelter (CC BY 4.0; https://creativecommons.org/licenses/by/4.0/), and Thomaz de Carvalho Callado (CC BY 4.0; https://creativecommons.org/licenses/by/4.0/).

In this study, we describe a striking feature of colorful feathers in some songbirds: Concealed beneath the colorful outermost layer of plumage, many birds have a highly reflective white or highly absorbent black layer of plumage formed by prominent white or black regions of individual contour feathers, located between the colorful tip and the downy base (Fig. 1A). We observed these white and black feather regions (hereafter achromatic regions) in male tanagers in the genus *Tangara* (family: Thraupidae), a group with exceptional color diversity that includes both barb-based noniridescent structural colors and carotenoid-pigmented plumage (Fig. 2). Notably, the color of the hidden achromatic feather layer in male tanagers varies with the color of the feathers on top. Carotenoid-pigmented plumage (red, yellow) is paired with a hidden white layer, while structurally colored plumage (blue, violet) is paired with a hidden black layer (Fig. 1B). Because the colorful tips of *Tangara* feathers that make up the outermost colorful layer of feathers are not opaque, we hypothesized that optical interactions between hidden achromatic layers of feathers and visible colorful layers of feathers have a substantial effect on the overall appearance of colorful plumage.

Pigmentary color depends on selective absorption of light by pigments and light scattering from surrounding tissue, rather than reflectance from the pigments themselves (*17*, *23*, *27*–*29*). Consequently, tissues containing carotenoids or other colorful pigments in a range of animals and plants often display specializations for maximizing light scattering (*30*–*33*), including air spaces that scatter light within the barbs of some yellow carotenoid-pigmented feathers (*27*, *34*). White feathers primarily scatter light. Therefore, we predicted that hidden white feather layers underneath carotenoid-pigmented layers scatter light back through carotenoid pigments, increasing the brightness of red and yellow plumage.

In contrast, barb-based noniridescent structural colors in avian feathers (such as the structural colors in *Tangara* tanagers) are produced by coherent scattering of light from keratin-air nanostructures within the ramus of the feather barb (*20*, *21*). Incoherent scattering of light from tissues surrounding the nanostructure can dilute the reflectance of the structural color and produce a pale, unsaturated color (*20*, *35*). Accordingly, barb-based structural colors in feathers generally contain a layer of melanin in the ramus which absorbs light that would otherwise be scattered by the surrounding keratin (*20*, *35*). This feature is similar to layers of melanin which co-occur with nanostructures in other colorful animal tissues (*8*, *36*, *37*). Black feathers primarily absorb light. Therefore, we predicted that hidden black feather layers absorb light that passes through structurally colored feather layers, increasing the saturation of blue and violet plumage.

We observed the presence of achromatic feather layers across males in the genus *Tangara*, where white layers are paired with carotenoid-pigmented plumage and black layers are paired with structurally colored plumage. We chose a subset of carotenoid-pigmented and structurally colored plumage patches to study in both female and male *Tangara* and documented the morphological features and reflectance of achromatic feather layers in these patches. We tested the predictions that white and black achromatic feather layers alter the brightness of carotenoid-pigmented and saturation of structurally colored plumage, respectively, using both experimental approaches and optical modeling. To understand the relevance of achromatic layers to the production of colorful signals, we assessed the relative contributions of achromatic and colorful layers to sexual dichromatism of colorful plumage patches. Last, we examined achromatic feather layers across passerine songbirds, including both colorful lineages with carotenoid pigments and structural colors and lineages which are restricted to melanin pigmentation.

## RESULTS

### White and black feather regions form a distinct, highly reflective or absorbent achromatic layer

We first comprehensively surveyed achromatic feather layers across six standard body regions in males from 26 *Tangara* tanagers (Materials and Methods). We found that carotenoid-pigmented plumage was always associated with an underlying white achromatic layer and structurally colored plumage was always associated with an underlying black achromatic layer in these male patches. We next selected six carotenoid-pigmented crown, rump, and belly patches and six noniridescent (barb-based) structurally colored crown, rump, and belly patches from across *Tangara* to study in detail (Fig. 2A and table S1) and then sampled contour feathers from those patches for females and males (Materials and Methods). We observed that individual feathers have three distinct regions: a colorful tip, an intermediate white or black achromatic region, and a plumulaceous downy base (Fig. 1A). The colorful tip and achromatic regions together generally comprise the pennaceous part of the feather (see the “Defining colorful, achromatic, and downy regions of feathers” section of the Supplementary Text for details and exceptions). When arranged on a bird’s body, white or black achromatic regions of individual feathers form a continuous white or black layer of plumage concealed under colorful layers (Fig. 1, B and C). In these individual feathers, we identified two exceptions to the general pattern that carotenoid-pigmented tips are paired with a white achromatic region and structurally colored tips are paired with a black achromatic region. First, we found that feathers from three of the six carotenoid-pigmented patches that we sampled in female tanagers had black achromatic regions, rather than white achromatic regions as in males. We expand on this result below (the “Sexual dichromatism of carotenoid signaling patches primarily arises from variation in the achromatic layer and not from the colorful layer” section). Second, we found that the achromatic regions switch from white to black, or black to white, at the edges of many plumage patches where there is a sharp transition from one type of color production mechanism to another (i.e., from structural color or melanin pigmentation to carotenoid pigmentation) (illustrated in Fig. 1B). In these cases, colorful feather tips from one patch rest on top of the achromatic regions of feathers from the adjacent, contrasting patch, and the achromatic region correspondingly switches in color so that the colorful tips always rest on top of the typical achromatic color for that mechanism. For example, while carotenoid-pigmented feathers sampled from the middle of a red or yellow patch have white achromatic regions, carotenoid-pigmented feathers sampled from the edge of a red or yellow patch have black achromatic regions when located next to a blue structurally colored patch or a black melanin-pigmented patch (Fig. 1, B and E, and fig. S14, A and C). Correspondingly, while structurally colored or melanin-pigmented feathers sampled from the middle of a patch have black achromatic regions, feathers sampled from the edges of the same patches have white achromatic regions when located next to a yellow or red carotenoid-pigmented patch (fig. S14B). The overall effect is that the boundaries of the hidden achromatic layer are aligned with the boundaries of the colorful layer above, suggesting that the achromatic layer has a precise optical function.

To demonstrate how the achromatic regions of individual feathers form white and black achromatic layers beneath colorful layers, we removed the colorful feather tips from the rump and crown in a *Tangara chilensis* specimen (Fig. 1C). We measured the reflectance of the exposed achromatic layers using hyperspectral imaging (HI; Materials and Methods). We found that achromatic feather regions form a continuous layer of white or black plumage that is highly reflective (white) or absorbent (black) (Fig. 1, C and D). The reflectances of white and black achromatic layers (~55% peak reflectance for white and 5% peak reflectance for black) (Fig. 1D) in the achromatic zone are comparable to reflectance values of white and black plumage patches across a wide range of birds (*18*, *38*, *39*).

### Achromatic feather layers enhance brightness of carotenoid-pigmented layers and saturation of structurally colored layers

We predicted that white achromatic layers increase the brightness of carotenoid-pigmented plumage and that black achromatic layers increase the saturation and decrease the brightness of structurally colored plumage. We expected that the hue of carotenoid-pigmented and structurally colored feathers would not be affected by achromatic layer reflectance, because hue should be controlled by the chemical structure of the pigment or specific features of nanostructures (*21*, *40*–*42*). We conducted a series of experiments to test these predictions. We placed carotenoid-pigmented and structurally colored feathers from both females and males on white and black surfaces and measured changes in brightness, saturation, and hue. We used several complementary methodological approaches to assess changes in coloration at different levels of feather organization, including ultraviolet-visible (UV-vis) microspectrophotometry (MSP) of individual feather barb rami and both multispectral photography (MP) and HI of entire colorful feather tips (Materials and Methods; see Fig. 2 for a summary of the experimental data collected). Our MSP and MP measurements used white and black photography standards with comparable reflectance to real white and black feathers as backgrounds (see fig. S1 for photography standard reflectances), while our HI measurements captured changes in reflectance of one carotenoid-pigmented and one structurally colored feather placed directly onto the exposed white and black achromatic layers from the *T. chilensis* specimen (see Fig. 1D for specimen achromatic layer reflectance). Our MSP and HI experiments yielded reflectance spectra from 300 to 700 nm and 400 to 700 nm, respectively, while our MP experiments yielded calibrated red, green, blue (RGB) images. For all reflectance spectra from our MSP and HI experiments, we calculated brightness (the mean reflectance across the spectral range), saturation (the contribution of the spectral peak to brightness), and hue (the wavelength of maximum reflectance). To calculate color metrics from multispectral images, we modeled colors in a cylindrical hue, saturation and lightness (HSL) color space and calculated lightness (the long axis of the cylinder, measured as a percent), saturation (the distance from the center to the edge of the cylinder, measured as a percent), and hue (the angle around the central axis of the cylinder, measured in degrees) (Materials and Methods). While measurements of brightness, saturation, and hue from reflectance spectra (MSP and HI) are analogous to measurements of lightness, saturation, and hue from MP images, the values are not directly comparable (Materials and Methods).

**Fig. 3.**
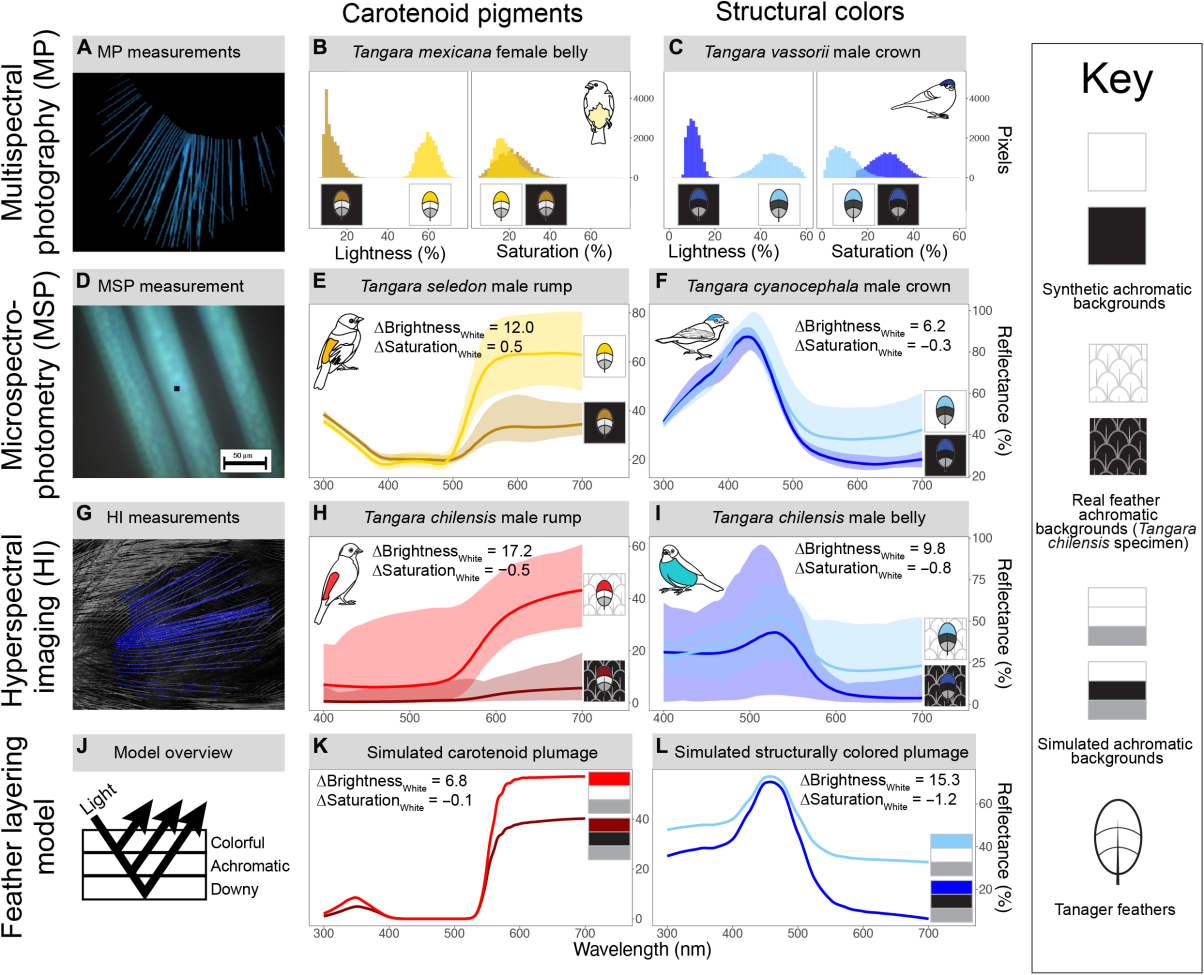
White achromatic backgrounds increase the brightness of carotenoid-pigmented feathers, and black achromatic backgrounds increase the saturation and decrease the brightness of structurally colored feathers. We experimentally measured changes in reflectance for carotenoid-pigmented and structurally colored feathers on white and black backgrounds with MP (**A** to **C**), MSP (**D** to **F**), and HI (**G** to **I**) and also used an optical model of colorful, achromatic, and downy feather layers to simulate the effects of achromatic background on carotenoid-pigmented and structurally colored plumage [(**J**), example simulations shown in (**K**) and (**L**)]. Carotenoid-pigmented feathers are brighter on white backgrounds [(B), (E), (H), and (K)], and structurally colored feathers are more saturated and darker on black backgrounds [(C), (F), (I), and (L)]. Shaded areas in (E) and (F) are the reflectance for nine barbs measured with MSP, and shaded areas in (H) and (I) are the reflectance of 500 subsamples from the HI ROI. Solid lines are the mean reflectance. Inset ΔBrightness and ΔSaturation values are the change in brightness or saturation on white backgrounds compared to black backgrounds. We chose to display results from individual species [(B), (C), (E), (F), (H), and (I)] to demonstrate the consistent optical effects of achromatic background on plumage coloration across a range of focal patches, but results from all other species and focal patches are similar and presented in figs. S4 and S5. We summarize changes in lightness/brightness, saturation, and hue on white and black backgrounds across all focal patches in Table 1 and list the full lightness/brightness, saturation, and hue values for every focal patch on white and black backgrounds in tables S2 and S3.

All three experimental approaches (MSP, MP, and HI) showed substantial increases in the brightness (MSP and HI) or lightness (MP) of carotenoid-pigmented feathers on white backgrounds (Table 1; Fig. 3, B, E, and H; tables S2 and S3; and figs. S4 and S5). In addition to these large changes in brightness, we observed minor increases in the saturation of carotenoid-pigmented feathers on white achromatic backgrounds measured with MSP and minor decreases when measured with both HI and MP (Table 1; Fig. 3, B, E, and H; tables S2 and S3; and figs. S4 and S5). The hue of carotenoid-pigmented feathers did not change substantively on white versus black achromatic backgrounds when measured with MP or HI and shifted for some feathers when measured with MSP (Table 1, tables S2 and S3, and figs. S4 and S5).

**Table 1.** Experimental changes in lightness or brightness, saturation, and hue of carotenoid-pigmented and structurally colored feathers on white achromatic backgrounds relative to black achromatic backgrounds. Values for MP and MSP experiments are mean ± SD across all samples. Because HI experiments were performed over a single carotenoid-pigmented and single structurally colored feather, these values lack an SD. Note that the “number of patches” includes both female and male patches for each of the six carotenoid-pigmented and six structurally colored focal patches.

Method	Number of patches	Feathers per patch	Mechanism	∆Lightness on white (%)	∆Saturation on white (%)	∆Hue on white (°)
MP	12	3	Carotenoid	39.1 ± 6.5	−7.7 ± 5.2	2.8 ± 1.6
MP	12	3	Structural	27.9 ± 5.9	−20.1 ± 2.7	1.9 ± 2.2
				**∆Brightness on white (%)**	**∆Saturation on white**	**∆Hue on white (nm)**
MSP	12	1	Carotenoid	14.1 ± 3.3	0.3 ± 0.2	125.6 ± 161.6
MSP	12	1	Structural	6.6 ± 2.5	−0.2 ± 0.1	6.8 ± 5.7
HI	1	1	Carotenoid	17.2	−0.5	0.0
HI	1	1	Structural	9.8	−0.8	1.0

All three experimental approaches showed increases in saturation and decreases in brightness (MSP and HI) or lightness (MP) for structurally colored feathers on black backgrounds compared to white backgrounds (Table 1; Fig. 3, C, F, and I; tables S2 and S3; and figs. S4 and S5). The hue of structurally colored feathers did not change substantively on white versus black achromatic backgrounds (Table 1, tables S2 and S3, and figs. S4 and S5).

Overall, our experimental results revealed that white achromatic layers increase the brightness and saturation of pigmented feathers, while black achromatic layers increase the saturation and decrease the brightness of structurally colored feathers. These effects are consistent across synthetic (MP and MSP experiments) and real feather achromatic backgrounds (HI experiments) and across different levels of feather organization (the entire colorful tip of the feather in MP and HI experiments or individual feather barbs in MSP experiments) (Table 1 and Fig. 3).

### Optical models and avian visual modeling replicate the experimental effects of achromatic background on brightness and saturation

A constraint of our experimental approaches is that we measured changes in reflectance for a single feather at a time. We observed that the colorful layer of plumage is only a single feather thick in many patches. However, colorful layers may include multiple overlapping colorful feather tips in other cases, which could make the colorful layer less transparent as a result of higher pigment concentrations, thicker plumage, or more reflective nanostructures. How consequential are the effects of achromatic layer reflectance or absorption on plumage color in these cases?

To answer this question, we built a model of total plumage reflectance that includes optical interactions among feather layers (Materials and Methods). We extended a multilayer color patch model that simulates the reflectance of a dermal chromatophore unit formed by layers of red and yellow pigmented cells (erythrophores and xanthophores, respectively), structurally colored cells (iridophores), and dark melanized cells (melanophores) (*43*). This model was originally developed to model color production in animals with dermal chromatophores (fish, reptiles, amphibians, and others), but the general features (distinct, contiguous layers of pigmented and structurally colored tissues) are similar to the colorful, achromatic, and downy layers of feathers that we identified in tanagers. We modeled optical interactions among colorful, achromatic, and downy feather layers and predicted total plumage reflectance across a wide range of variation in pigments and nanostructures (Materials and Methods).

Consistent with our experimental results, our simulations revealed that white achromatic layers increase the brightness of carotenoid-pigmented plumage (Fig. 3K and fig. S8A). These simulations additionally showed that the magnitude of these effects is consistent across variation in simulated pigment optical density (fig. S8A). The saturation of simulated carotenoid-pigmented plumage was slightly lower on white achromatic backgrounds, but the magnitude of these effects was small, and achromatic layer reflectance has no effect on hue (Fig. 3K and fig. S8, D and G).

Our simulations of structurally colored plumage revealed that black achromatic layers increase total plumage saturation and decrease total plumage brightness without changing hue (Fig. 3L and fig. S8), consistent with experimental results. We found that achromatic layer reflectance affects structurally colored plumage across the range of simulated variation in nanostructures, but the effects of achromatic layer reflectance are highest when the reflectance from the nanostructures themselves is low (fig. S8, B and E) or when the color produced by the nanostructure is highly saturated (fig. S8, C and F).

Last, we used avian visual modeling to calculate the effects of achromatic background on the brightness and saturation of our simulated spectra from the avian visual perspective (Materials and Methods). Birds have tetrachromatic color vision, and many birds, including tanagers, have visual sensitivity that extends into UV wavelengths (*44*, *45*). We were constrained in our ability to measure UV reflectance in our MP and HI experiments (see Materials and Methods for details), but we simulated spectra across the full range of wavelengths visible to birds (300 to 700 nm). We first plotted our simulated spectra in an avian tetrachromatic color space that represents the relative stimulation of the four avian color cones (fig. S9B) (*46*). For every simulated spectrum, we calculated luminance (the stimulation of the avian double cone, a measure of brightness) (*47*) and chroma (the distance from each point to the achromatic center of the tetrahedron, a measure of saturation). We found that white achromatic backgrounds increase the luminance of carotenoid-pigmented plumage and black achromatic backgrounds increase the chroma of structurally colored plumage (fig. S9C), consistent with experimental results.

### Sexual dichromatism of carotenoid signaling patches primarily arises from variation in the achromatic layer and not from the colorful layer

In *Tangara* tanagers, females and males both have ornamented, colorful plumage, but colors tend to be duller in females (*48*). We used spectrophotometry of female and male museum specimens to measure the reflectance of each of the six carotenoid-pigmented and six structurally colored patches in our dataset and ultimately determine the extent of sexual dichromatism in each patch (Materials and Methods). These reflectance spectra account for interactions between all layers of feathers that make up a patch (colorful, achromatic, and downy). We first measured brightness and saturation from reflectance spectra and compared these values between females and males. We next assessed differences in female and male color from the avian visual perspective by modeling female and male spectra in avian tetrahedral color space and calculating the noise-weighted Euclidian chromatic and luminance distances (∆*S* and ∆*L*, respectively) between female and male color for each of the patches in our dataset (Materials and Methods). These chromatic and luminance distances are based on the receptor noise-limited model (*49*) and are used to estimate whether two color signals are discriminable for a particular visual system, with values higher than one being theoretically discriminable, but values of 2 to 3 considered a more reliable threshold of a discriminable distance (*50*, *51*).

Next, to determine whether measured differences in coloration between females and males are due to colorful or achromatic feather layers, we examined individual feathers sampled from each female and male patch. We characterized the size and reflectance of achromatic feather regions using MP and compared these features between females and males. Last, we measured the size of colorful feather regions from MP images and the reflectance of colorful barbs using MSP and compared these features between females and males (Materials and Methods; see Fig. 2 for an overview of empirical data collection).

**Fig. 4.**
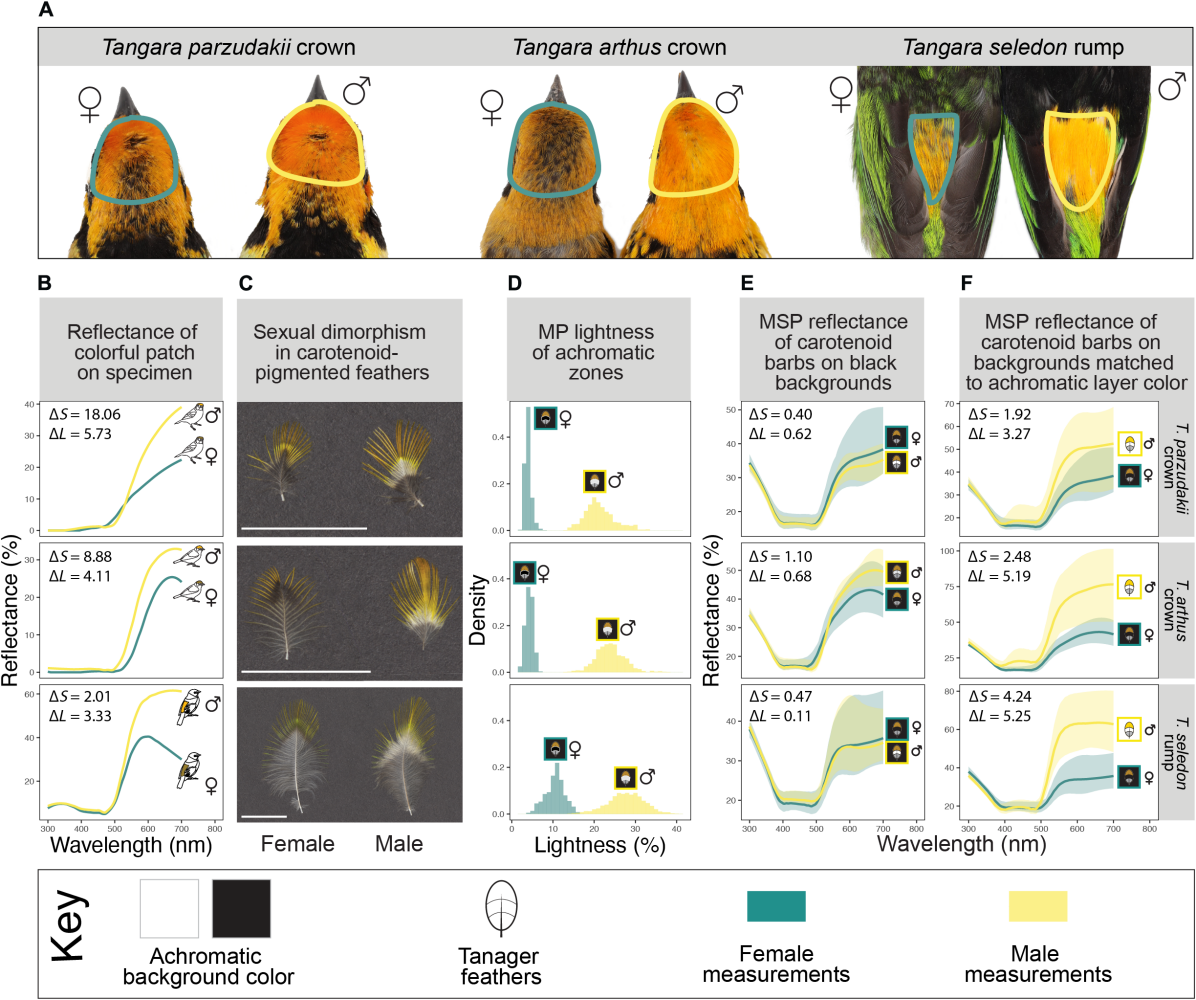
Sexual dichromatism in three carotenoid patches is primarily the result of dichromatism in achromatic layers and not variation in pigments. Photographs in (**A**) show each of the patches on female (left) and male (right) specimens. (**B**) Reflectance of each female and male plumage patch measured on specimens with spectrophotometry. These measurements capture the reflectance of the entire feather stack (colorful, achromatic, and downy layers) shown in (A). (**C**) Sexual dichromatism in individual feathers from each of these plumage patches, with females lacking a prominent white achromatic region. Scale bars, 1 cm. (**D**) Lightness of female and male feather achromatic regions measured with multispectral imaging on black backgrounds and plotted as a density histogram. To understand whether dichromatism at the level of the patch in (B) was produced by colorful or achromatic layers of feathers, we first compared coloration in females and males measured on the same black background with MSP (**E**) and then measured male and female feathers on backgrounds corresponding to their achromatic layer colors (**F**). Inset values in (B), (E), and (F) are chromatic (∆*S*) and luminance (∆*L*) contrasts between female and male spectra with higher values corresponding to higher estimated discriminability according to avian visual models. Spectra in (B) are the average of three repeated measurements with a spectrophotometer. Shaded areas in (E) and (F) correspond to nine barbs measured with MSP, and solid lines are the mean reflectance. From left to right, specimens imaged (A) are LACM 29403, LACM 29405, LACM 29397, LACM 83347, LACM 28602, and LACM 28114.

We found large differences between female and male coloration in three yellow carotenoid patches (*Tangara arthus* crown, *Tangara parzudakii* crown, and *Tangara seledon* rump), as determined by the disparities between female and male brightness and saturation measured from spectra and ∆*S* and ∆*L* values above 2 (Fig. 4B and table S4). We also found moderate differences in brightness and saturation in *Tangara icterocephala* belly, with correspondingly high ∆*S* (but not ∆*L*) values (table S4). Differences in female and male color were more subtle in the remaining two carotenoid patches (table S4 and fig. S10). Unexpectedly, in the three most dichromatic carotenoid-pigmented patches, we observed a deviation from the general trend that carotenoid-pigmented feathers are paired with white achromatic regions: While males had yellow tips paired with bright white achromatic regions, females had yellow tips paired with black achromatic regions (Fig. 4, C and D).

To determine whether the observed differences in achromatic feather layers in *T. arthus*, *T. parzudakii*, and *T. seledon* could explain the dichromatism at the level of the entire patch, we first compared the reflectance spectra of individual female and male yellow feather barbs measured on black backgrounds with MSP. We found that coloration in the yellow feather barbs is not appreciably different between females and males (Fig. 4E), and female and male carotenoid spectra are not predicted to be discriminable in avian visual space (∆*S* and ∆*L* < 1) when measured on the same color achromatic background (Fig. 4E and table S4). The similar reflectance spectra between female and male barbs suggest that differences in carotenoid concentration in the colorful feather region are modest and do not explain the dichromatism observed at level of the patch (Fig. 4B). Females have carotenoid pigmentation in a smaller proportion of the feather than males (Fig. 4C and fig. S11), which could potentially lead to a less continuous pigmented feather layer; in these female patches, the effect is that the achromatic layer is less hidden (Fig. 4A). We next compared the MSP spectra of yellow female feathers on black backgrounds and yellow male feather barbs on white backgrounds, consistent with the observed colors of female and male achromatic layers, respectively (Fig. 4F). We found that accounting for variation in achromatic layer color produces dichromatic reflectance spectra that are similar to reflectance spectra measured on a specimen (Fig. 4B), and the associated chromatic and luminance distances predict that these spectra are likely discriminable from the avian visual perspective (Fig. 4F). Overall, these results suggest that differences in total female and male coloration are due primarily to variation in the color of the achromatic layers rather than differences in the concentration of carotenoid pigment deposited in feather barbs. Furthermore, dichromatism is likely enhanced by differences in the relative size of achromatic and pigmented regions: Females have a smaller pigmented proportion of the feather, meaning that the achromatic layer is more prominent and directly visible (Fig. 4, A and C).

For structurally colored patches, we found evidence of sexual dichromatism primarily in the crown in *Tangara vassorii* and the belly of *T. chilensis*, as determined by differences in brightness and saturation measured from spectra and ∆*S* and ∆*L* values (table S4). We did not find pronounced sexual dichromatism (i.e., white versus black) in the hidden achromatic layers, which were black in both female and male structurally colored plumage (fig. S10B). However, we found differences in the darkness of feather achromatic regions in several patches, and darker achromatic layers were associated with darker coloration at the level of the patch (fig. S10). These effects were most pronounced in the crown and rump patches of *T. vassorii*, where males had substantially brighter plumage and brighter achromatic regions than females (table S4 and fig. S10, A and B). A larger proportion of the feather was structurally colored in male *T. vassorii* crown and rump feathers (fig. S11), and in the rump, males had smaller achromatic regions but larger, downier feathers overall (fig. S12).

### Hidden achromatic layers of feathers have evolved in distantly related, colorful passerine birds

Tanagers are part of a radiation of songbirds called passerines (order: Passeriformes). As a group, passerines have enormous color diversity, including many lineages whose plumage displays carotenoid pigmentation and/or structural coloration but also lineages restricted to melanin pigmentation (*22*, *52*–*54*). We used confocal microscopy and photography (Materials and Methods) to document coloration in hidden feather layers in distantly related passerine lineages, including both lineages that have evolved carotenoid pigmentation and/or noniridescent structural colors (for example, manakins, cotingas, and fairy wrens) and lineages with only melanin pigmentation (for example, ovenbirds and antbirds). Together, we examined the color of hidden feather layers in 34 patches from 27 specimens representing 16 passerine families (table S5).

**Fig. 5.**
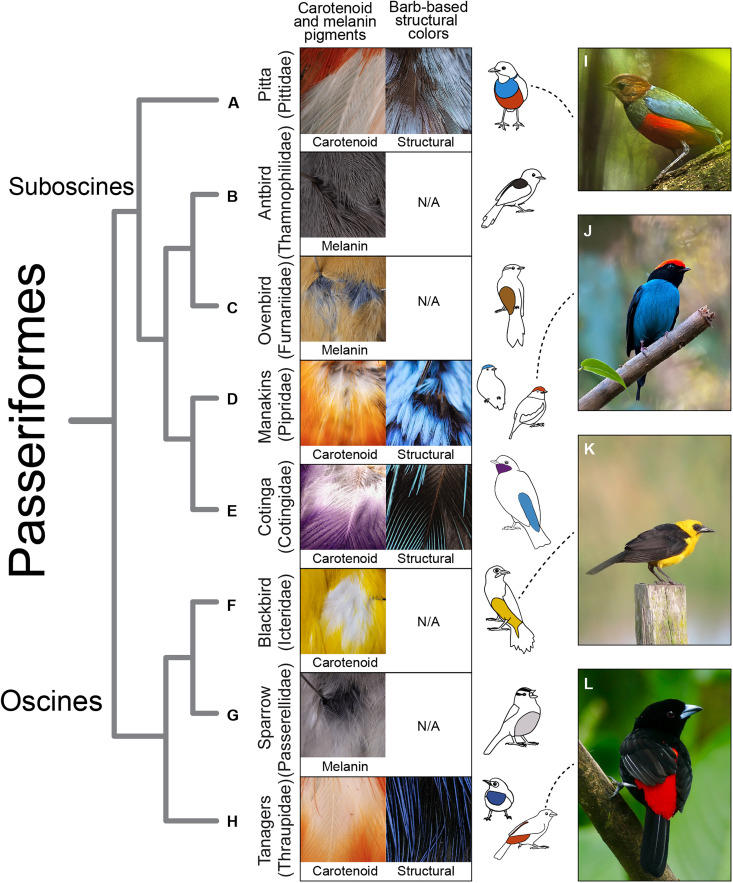
White and black achromatic feather layers have evolved across colorful passerine birds. We examined hidden feather layers in lineages with carotenoid pigments (**A**, **D**, **E**, **F**, and **H**) and noniridescent structural colors [(A), (D), (E), and (H)]. We compared them to lineages that have melanin pigments in their feathers but lack carotenoids and structural colors (**B**, **C**, and **G**). Distinct white and black achromatic layers are present underneath carotenoid-pigmented and structurally colored feathers across passerines but absent from eumelanin (B) and pheomelanin-pigmented (C) feathers. Illustrations in (A) to (H) show body regions imaged for each bird. In (**I**) to (**L**), we highlight several examples of colorful passerines we examined. (A) Confocal microscope (left) and macro image (right) of *Erythropitta erythrogaster* (LACM 66188, sex unknown). (B) Macro image of *Cercomacroides nigrescens* (LACM 32003, male). (C) Macro image of *Dendroma rufa* (LACM 27280, female). (D) Macro images of *Chiroxiphia caudata* (left, LACM 94018, male) and *Lepidothrix coronata* (right, LACM 36522, male). (E) Confocal images of *Cotinga amabilis* (LACM 77881, male). (F) Macro image of *Gymnomystax mexicanus* (LACM 39278, sex unknown). (G) Macro image of *Zonotrichia leucophrys* (LACM 111863, female). (H) Confocal images of *Ramphocelus passerinii* (left, LACM 14648, male) and *Dacnis berlepschi* (right, LACM 37455, male). N/A labels indicate that a particular lineage lacks structural colors in the barb ramus. Phylogenetic relationships shown are following a passerine tree based on genomic data (*83*) with arbitrary branch lengths. Lineages shown above are a subset of the passerines we examined, which are fully documented in table S5 and fig. S13. Exceptions to the pairing of white achromatic layers with carotenoids and black achromatic layers with structural colors in passerines are shown in figs. S15 and S16. Image credits: (I) Francesco Veronesi (CC BY-SA 2.0), (J) Dario Sanches (CC BY-SA 2.0), (K) Félix Uribe (CC BY-SA 2.0), and (L) Bernard Dupont (CC BY-SA 2.0). Information for CC BY-SA 2.0 license can be found at https://creativecommons.org/licenses/by-sa/2.0/deed.en.

We found that passerines with feathers colored exclusively by melanin pigments do not have distinct achromatic layers under melanin-pigmented feathers (Fig. 5, B, C, and G, and table S5). In these birds, individual contour feathers generally contain two distinct regions: a black, brown, or gray melanin-pigmented pennaceous tip and a gray plumulaceous base. As a result, the hidden layers of plumage tend to be the same color as the outermost visible layer, although there is some variation in the intensity in melanin pigmentation along the length of the pennaceous tip (Fig. 5, B, C, and G). While white achromatic layers underneath pheomelanin-pigmented plumage could theoretically increase the brightness of the rusty red/brown colors produced by pheomelanin pigments, we did not observe this pairing in our survey of passerines.

In contrast, we found that distinct white achromatic layers are present beneath carotenoid-pigmented plumage and black achromatic layers are present beneath structurally colored plumage in many distantly related colorful passerines (Fig. 5, A, D, E, F, and H; table S5; and fig. S13), and feathers from these colorful patches have three distinct regions (colorful, achromatic, and downy). Many of these lineages appear to be under intense sexual and social selection for colorful signals, including polygynous lineages with elaborate courtship displays such as manakins (*55*, *56*) and cotingas (*57*). In several of these species, we also observed changes in the color of the feather achromatic region from white to black or black to white at the intersections of contrasting plumage patches (fig. S14, D to H), similar to the phenomenon we observed in *Tangara*. We observed one case of dark red carotenoid-pigmented plumage paired with black achromatic layers and two cases of pale blue structurally colored plumage paired with white achromatic layers (fig. S15). These examples suggest that optical interactions among hidden achromatic and visible colorful layers may be leveraged to produce a wide range of optical effects—not just the bright carotenoid-pigmented and saturated structurally colored plumage that we identified in tanagers. Distinct achromatic layers are not ubiquitous in colorful birds, however, and we observed several colorful plumage patches where feathers appear to lack achromatic regions completely (fig. S16). We document these exceptions in *Supplementary text: Variation in achromatic layers in colorful passerines*, table S5, and figs. S15 and S16. Overall, our survey of passerine feather layers suggests that highly reflective or absorbent achromatic layers may be a derived feature of passerines with carotenoid pigmentation and structural coloration and may evolve in response to sexual and social selection for bright and saturated plumage coloration.

## DISCUSSION

In this study, we showed that *Tangara* tanagers—and a host of other vibrantly colored songbirds—have a layer of achromatic plumage hidden under the outermost colorful feather layer that has major optical consequences for the appearance of colorful plumage—and ultimately its perception in sexual and social signaling contexts. Despite longstanding and intense research interest in the function and evolution of plumage color in birds, the potential for optical interactions among visible and concealed layers of feathers has largely been neglected. Consequently, the presence and importance of hidden achromatic feather layers have been overlooked. In tanagers, the systematic variation in achromatic layer color (i.e., between white and black) according to visible layer color—as well as the extraordinary coordination of achromatic regions in feathers that span contrasting plumage patches—suggests that achromatic layers are shaped by selection for their optical functions. Thus, avian plumage is simultaneously stunningly beautiful and deeply complex: Creating the final product requires the coordination of light absorption and scattering via pigments and nanostructures not only within feather barbs and barbules but also across individual regions of entirely separate feathers that overlap on the body. It is therefore critical to study plumage coloration not only at the scale of the nanostructures and pigments that are present within the visible tip of the feather but also at the scale of interacting layers of visible and concealed feathers. By demonstrating this feature of avian coloration, we hope to invite a closer look at hidden layers of feathers in a wide range of birds, which can ultimately provide fresh insights into how colorful plumage functions and evolves across birds.

One line of insight to come from studying hidden feather layers involves the origins and evolutionary significance of differences in female and male coloration. We found that sexual dichromatism in several carotenoid-pigmented patches in *Tangara* is primarily the result of variation in achromatic layers, and differences in the color produced by carotenoid pigments in female and male feather barbs are unexpectedly modest. Carotenoid pigments are widely considered to be honest indicators of quality (*58*), and links between individual variation in red and yellow plumage and success in mating or other social interactions are frequently interpreted under honest signaling frameworks (*14*, *59*). A key assumption in this interpretation is that variation in red and yellow signals is produced primarily by differences in carotenoid pigmentation among individuals, which does not appear to be wholly the case between female and male *Tangara* tanagers. How generalizable are these results outside of tanagers? In contrast to many groups of colorful birds, both sexes are ornamented and look relatively similar in *Tangara* tanagers. In many avian systems, however, sexual dichromatism is more pronounced and likely involves considerable variation in both colorful and achromatic feather layers. However, we identified prominent white achromatic layers associated with carotenoid-pigmented plumage in males of many colorful and sexually dichromatic passerines (Fig. 5 and fig. S13), suggesting that achromatic layers likely play a large role in generating phenotypic variation in behaviorally important signals in birds beyond tanagers. These results demonstrate that, at the very least, interpreting the evolutionary significance of carotenoid signals is more complicated than is often assumed. Highly reflective white plumage may itself signal quality, although an experimental test in American goldfinches (*Spinus tristis*) found no links between white structural color production and food availability (*34*). An alternate possibility is that colorful signals produced by both colorful and achromatic layers of feathers may be shaped more by receiver biases or selection for efficient, conspicuous signals than selection for signal content. Further mechanistic, evolutionary, and behavioral study on the relevance of hidden achromatic layers to sexual and social signaling will help address these questions.

Achromatic layers of feathers are also likely shaped by selection for nonsignaling properties, and a second promising area of research is investigating how selection for nonsignaling functions of hidden feather layers may ultimately constrain or facilitate the evolution of vibrant coloration. Black plumage is associated with protection from solar radiation, resistance to bacterial degradation, increased mechanical strength, and heat retention (*17*, *60*). White plumage is associated with high reflectance in near-infrared wavelengths, which may affect thermoregulatory capacities (*61*). Selection for these nonsignaling functions in hidden layers of feathers may trade off with selection for coloration in some cases but be complementary in others. For example, selection for protection from solar radiation or bacterial degradation may be consistent with the production of saturated structurally colored plumage via black achromatic layers but conflict with the production of bright carotenoid signals via white achromatic layers. These tradeoffs may explain why females, but not males, have black achromatic layers paired with yellow carotenoids on several dorsal body patches in *Tangara*; in this case, the dichromatism in achromatic layers that we found in these three patches may be the result of sex-specific selection pressures for conspicuous plumage in males and crypsis or nonsignaling functions (such as protection from solar radiation) in females.

The patterns of dichromatism in achromatic and colorful feather regions we observed in *T. vassorii*—a species which inhabits higher elevations than any other member of the genus (*62*)—also hint at the existence of complex interactions among different feather functions. In this species, both females and males have blue structurally colored plumage paired with an underlying black achromatic layer. In females, however, the achromatic regions are larger and darker than in males (figs. S10 to S12). The optical consequence of the larger, darker achromatic region is darker blue plumage in females—yet the larger melanized region of female feathers may provide a greater thermoregulatory benefit to females than to males. Notably, males have much longer, downier feathers than females, a pattern which we did not observe in other species. Longer, downier feathers are associated with higher-elevation habitats in many birds (*63*). These patterns suggest that selection for thermoregulation at high elevations may interact differently with selection for signaling in *T. vassorii* females and males, producing dimorphism in downy, achromatic, and colorful feather layers. While an example from a single species is preliminary and these explanations are necessarily speculative, these patterns highlight how considering both the signaling and nonsignaling properties of achromatic feather layers may help explain inter- or intraspecific variation in coloration.

Last, the evolution of achromatic layers may also interact with the evolution of melanized skin underneath feathers, a trait which is present in roughly 5% of birds and is associated with protection from UV radiation (*64*). The evolution of melanized skin is predicted to relieve feathers from selective pressures related to UV radiation, potentially facilitating the evolution of white visible plumage (*65*)—or, by similar logic, the evolution of prominent white achromatic layers in species with carotenoid pigmentation. Testing these predictions will require more complete characterization of achromatic layers across diverse sets of species, but this line of research is likely to be informative. Overall, accounting for the thermoregulatory and other functional implications of hidden achromatic layers may help explain variation in plumage color or sexual dichromatism across latitudinal (*66*), elevational (*67*), or other ecological gradients.

Feathers are not opaque, and the tendency of plumage spectra to change when feathers are measured on different color backgrounds has been previously noted as a technical challenge for spectrophotometry (*68*). Here, we demonstrate that the pairing of translucent carotenoid-pigmented and structurally colored feather layers with white and black hidden feather layers is an evolved strategy to modulate the appearance of colorful plumage in birds. While many questions remain about the evolution of hidden achromatic layers in birds, optical interactions among contiguous colorful and achromatic layers of feathers appear to be crucial for producing and modulating plumage color. Although this phenomenon is underexplored in the context of avian plumage, the production of color via layers of scattering elements (structural colors) and absorbers (pigments) has clear parallels to other animal and plant systems, including other avian tissues (*23*). For example, colorful bare skin in birds is produced by structurally colored dermal collagen underlain by melanin pigments or by carotenoid pigments combined with dermal collagen arrays which themselves scatter long wavelengths, producing saturated orange and red colors (*69*). Outside of birds, layers of structurally colored and pigmented chromatophores interact to produce color in a wide range of fish, amphibians, reptiles, and cephalopods (*43*, *70*–*72*), while colorful pigments are paired with light-scattering specializations in flower petals (*33*) and structural colors are paired with layers of melanin in butterflies (*36*, *37*), spiders (*8*), and mammalian skin (*73*). Overall, interactions between structural colors and pigments are increasingly being recognized as an integral feature of color production in animals and plants (*23*, *74*), and further undescribed examples of structure-pigment interactions are likely extensive. Exploring structure-pigment interactions in animal and plant tissues may stimulate the development of new technologies in the future. For example, paired scattering and absorbing elements are commonly used to create colorful synthetic materials, in some cases directly inspired by bird feathers (*75*, *76*). As other authors have suggested, further study of structure-pigment interactions in nature could lead to bioinspired designs in areas as diverse as the development of nontoxic alternatives to common dyes or advances in light-harvesting technologies (*74*). Last, the close pairing of absorbing and scattering elements in colorful animal and plant tissues has a notable resemblance to many art forms. For example, painters applying a gesso primer (*69*), makeup artists, and glass artists strategically use hidden white layers to enhance colorful layers on top, an approach that the artist Dale Chihuly compellingly refers to as including a “cloud layer” in many of his glass sculptures. The study of color production in avian plumage and beyond continues not only to yield insights into evolutionary processes but also to inspire broader connections to art and bioinspired design.

## MATERIALS AND METHODS

### Initial observations of achromatic layers, patch selection, reflectance spectrophotometry, and feather sampling

To determine the extent of the achromatic layer across *Tangara*, we examined males from 26 of 27 species in the genus using museum specimens from the collections of the Natural History Museum of Los Angeles County (Los Angeles, CA; database acronym LACM) and the Academy of Natural Sciences of Drexel University (Philadelphia, PA; database acronym ANSP). For each specimen, we chose six standard patches (crown, rump, back, belly, breast, and epaulet). We classified each patch by color production mechanism and observed the color of the achromatic region (see the “Classifying *Tangara* patches by color production mechanism and selecting focal patches” section of the Supplementary Text for further details).

We next selected a subset of carotenoid-pigmented and structurally colored patches from across *Tangara* to focus on for further detailed study (Fig. 2A). We chose patches that were unambiguously either carotenoid pigmented or structurally colored because the optical effects of achromatic layer reflectance on colorful feathers that contain both carotenoids and nanostructures are likely to be complex; we nonetheless observed that plumage patches colored by both pigments and nanostructures are typically (but not exclusively) associated with black achromatic layers (see the “Classifying *Tangara* patches by color production mechanism and selecting focal patches” section of the Supplementary Text for further details). We chose six structural patches (two crown, two rump, and two belly) and six carotenoid patches (two crown, two rump, and two belly) spread across 9 of the 27 species in the genus *Tangara* and studied these patches in females and males (Fig. 2A and table S1).

We measured specimen patch color using UV-vis reflectance spectrophotometry. We used either OceanInsight (Orlando, FL) OceanOptics USB2000 or Flame UV-vis spectrophotometers with an OceanInsight PX-2 pulsed xenon light source, a 99% white Spectralon standard (Labsphere, North Sutton, NH, USA), and a black velvet dark standard. We took reflectance measurements at a distance of 5 mm from the plumage surface with the probe at 90°. We set integration time to 100 ms and boxcar width to five and took five scans to average. We measured each plumage patch three times and averaged the three spectra to account for measurement variation using pavo v. 2.8.0 (*77*) in R v. 4.2.0 (*78*).

We next sampled three individual feathers from each of the 12 focal patches for females and males. We also opportunistically sampled feathers from the intersections of patches with contrasting colors (table S1). We cleaned feathers with an ethanol wash before proceeding with imaging.

In addition to sampling individual feathers, we used embroidery scissors to remove colorful feather tips from the red rump and green crown of a male *T. chilensis* specimen (LACM 85951) to expose the white and black achromatic layers underneath (see the “Classifying *Tangara* patches by color production mechanism and selecting focal patches” section of the Supplementary Text for further details on specimen choice).

### Multispectral photography

To measure the effect of background color on reflectance of colorful tips, we photographed every feather in our main dataset (*N* = 72, including three feathers from each of the 12 patches we sampled for females and males; Fig. 2B) on white and black squares from a Calibrite ColorChecker Classic photography standard (Calibrite LLC, Wilmington, DE, USA) that has reflectance comparable to real achromatic layers in tanagers (the “Reflectance of Calibrite color backgrounds” section of the Supplementary Text). Photographs were taken in RAW format using a Nikon D7000 camera (modified to full-spectrum sensitivity) with a Nikkor 105-mm lens and a filter that allowed only visible light to enter the camera (Baader UV/IR-Cut/L filter: transmission, 420 to 680 nm). We were unable to take UV images to complement these visible spectrum images, because switching between camera filters resulted in minor adjustments to camera position that made aligning UV and visible spectrum images of very small feathers infeasible. Feathers were positioned inside of a custom light diffuser (spectrally flat polytetrafluoroethylene) with a microscope coverslip placed over the base of the feather to reduce feather curvature and ensure that the colorful tip was flush with the achromatic background. Feathers were illuminated with an ExoTerra Sunray 50W UV lamp (Hagen Inc./Exo-Terra, Montreal, Quebec, Canada). We took images of 99% white and 2% black Spectralon standards (Labsphere, North Sutton, NH, USA) using an identical setup and camera settings. These images were used to normalize images of feathers.

We generated multispectral images using the “photo screening” tool from the Multispectral Image Calibration and Analysis (MICA) toolbox (*79*) in ImageJ v. 1.53 (*80*). For each image, we measured reflectance of the corresponding white and black standard. MICA then produced a multispectral image which accounts for differences in illumination among photographs as well as correcting for differences in camera responses across channels (RGB). We then converted each resulting reflectance stack to an RGB image using the “Stack to RGB” function in ImageJ. Next, we manually selected a region of interest (ROI) of the colorful tip that included the top two-thirds of each colorful barb, which is representative of the portion of the colorful feather that is exposed when feathers are layered on specimens. We then exported each ROI as a PNG file, which contained an image of colorful barbs on white or black backgrounds. To sample only feather pixels and exclude background pixels, we used a custom RShiny app. In the app, we first converted images to a cylindrical HSL color space using the “RGBtoHSL” function in the R package imager (*81*). In HSL space, lightness is the height of the cylinder, measured as a percent; saturation is the distance from the center to the edge of the cylinder, measured as a percent; and hue is the angle around the central axis of the cylinder, measured in degrees. Lightness varies between 0 (black) and 100 (white), saturation varies between 0 (gray) and 100 (fully saturated), and hue varies from 0° to 360°. We used our app to interactively adjust hue, saturation, and lightness thresholds until the white or black background pixels were masked out and the remaining pixels corresponded only to colorful feather barbs. For each feather on each background (white or black), the outputs were distributions of hue, saturation, and lightness values for pixels in the colorful part of the feather. To summarize our experimental results, we combined the distributions of HSL values for each of the three feather replicates on each background from each patch (Fig. 3, B and C, and fig. S5). We then calculated the mean lightness, saturation, or hue for the combined distributions of HSL values on white and black backgrounds (table S2). Last, we calculated the mean lightness, saturation, and hue on white and black backgrounds for all carotenoid-pigmented feathers and all structurally colored feathers (Table 1).

We also used our multispectral images to measure the reflectance of achromatic regions (Fig. 2B). We used images of achromatic regions on contrasting backgrounds (white achromatic regions on black and black achromatic regions on white). We first selected the achromatic region of each feather from the appropriate image as an ROI (see the “Extended methods” section of the Supplementary Text for further details on how we defined the boundaries of achromatic regions). Because the structure of feathers in achromatic regions was complex and consisted of many overlapping barbs and barbules, we did not use our RShiny app to manually remove white and black background pixels as above. Instead, we exported distributions of RGB values for the entire PNG of the feather achromatic region on the contrasting white or black background and converted RGB values to HSL using the RGBtoHSL function in imager. We separately measured lightness values from images of the white and black background squares without a feather present and found that white Calibrite achromatic squares had lightness values above 60%, while black Calibrite achromatic squares had lightness values below 10%. We then removed pixels matching these background lightness values from the HSL distributions of the feather achromatic zones. For patches in our dataset where female and male feathers had dichromatic achromatic regions (for example, white in males and black in females, as was the case for three carotenoid-pigmented patches), we were not able to apply this approach, because comparing the reflectance of a white achromatic zone on a black background to a black achromatic zone on a white background would be misleading. Instead, for each of these patches, we selected images of both female and male feathers on black backgrounds and directly measured the lightness of the achromatic regions by drawing lines along barbs in the achromatic region and extracting HSL values for every pixel in that line using the “RBG Profiles” tool in ImageJ. To compare the lightness of achromatic regions while accounting for differences in their relative sizes (and therefore differences in the number of pixels in MP image) between sexes and among patches, we plotted distributions of lightness values as density histograms. We found one carotenoid-pigmented patch where feathers from both sexes had a mix of white and black or absent achromatic zones (*T. icterocephala* belly). We compared the reflectance of the white regions from two feathers and discarded measurements from the feather with a black achromatic zone (females) or lacking an achromatic zone (males).

### Microspectrophotometry

We used a CRAIC UV-vis microspectrophotometer (Chapel Hill Nanofabrication and Analytical Laboratory, Chapel Hill, NC) to measure changes in reflectance from 300 to 700 nm for individual feather barbs on white and black backgrounds. For each of the 12 plumage patches in our dataset, we chose a single feather from females and males (*n* = 24 feathers total; Fig. 2B) and placed each feather on the white and black sections of a Calibrite Color Checker 3-step Grayscale. We placed a coverslip over the base of each feather to ensure that it was flat against the background. We set scanning time to 201 ms and scans to average to 60 and took measurements at ×40 magnification. We took measurements from nine colorful barb rami from across the colorful tip of the feather. For each barb, we measured halfway along the colorful part of the barb (i.e., midway from where the barb became colorful to the tip of the barb). We calibrated with a 99% white Spectralon standard and a black velvet dark standard and took new standard readings after each male-female feather pair.

We processed spectra and extracted color measurements using pavo. We smoothed spectra using the function procspec() with span set to 0.3. We calculated summaries of color using summary() and extracted brightness (the mean relative reflectance over the measured range of wavelengths; *B2* in pavo), saturation (the wavelength of maximum reflectance minus the wavelength of minimum reflectance, divided by mean relative reflectance; *S8* in pavo), and hue (the wavelength of maximum reflectance; *H1* in pavo). In addition to measuring reflectance, we used the microspectrophotometer to measure the transmission of light through colorful and achromatic feather regions and used these results to parameterize our optical modeling (see the “Extended methods” section of Supplementary Text for details on transmission measurements and optical modeling).

### Hyperspectral imaging

We used HI to measure the reflectance of white and black achromatic layers on a *T. chilensis* specimen with colorful feather tips removed (Fig. 1C), as well as the reflectance of individual *T. chilensis* feathers placed on these exposed white and black achromatic layers (Fig. 2B). To collect these feather color data, we placed a red carotenoid-pigmented (*T. chilensis* rump) or blue structurally colored (*T. chilensis* belly) feather on the exposed white (rump) and black (crown) *T. chilensis* achromatic layers. We used embroidery thread to position each feather so the colorful tip was resting flush to the exposed achromatic layer. In summary, we collected six hyperspectral images: one of the exposed white layer in the rump, one of the exposed black layer in the crown, and one each containing the red or blue feather on each of these achromatic backgrounds.

We collected all hyperspectral images using a Resonon (Resonon, Bozeman, MT) Pika NUV hyperspectral imager with a Nikkor 105-mm lens, which we chose to accommodate the small size of feather samples. We illuminated samples with broad-band light produced by a 450W Xenon ozone free arc lamp (Newport Corp., Irvine, CA). We mounted the hyperspectral camera 52 cm above a linear translation stage that moved the specimen across the field of view of the camera, capturing exposures at a rate of 5 frames per second, with an integration time of 200 ms and a gain of 6 dB. In this configuration, the imager was sensitive between 400 and 800 nm; below this range, readings were dominated by noise. Each hyperspectral image was spatially and spectrally calibrated with respect to a 99% Spectralon (Labsphere, North Sutton, NH) reflectance standard. The camera settings, data collection, and the linear translation stage were controlled with Resonon SpectrononPro software. Further details of the HI process are provided elsewhere (*82*).

To analyze reflectance in these hyperspectral images, we manually produced (using MATLAB, MathWorks, Natick, MA) polygonal ROIs from which to sample reflectance spectra. For the achromatic layers, the ROI encompassed the whole exposed patch, and for the feathers, the ROIs contained only the colorful feather barbs. For each image, we then randomly generated 500 sample locations from the ROI, and at each sample location, we extracted the median of a 3 × 3 pixel area (see Fig. 3G for an example ROI with samples). These samples were used as the data for further analysis. We used pavo to smooth spectra, calculate the mean reflectance across all 500 samples from each hyperspectral image, and extract measures of brightness, saturation, and hue from the mean spectrum as described in the “Microspectrophotometry” section above.

### Optical modeling of feather layers and avian visual modeling of simulated spectra

We adapted a model of multilayer color production based on the dermal chromatophore unit (*43*) to model total patch reflectance as a function of optical interactions among colorful, achromatic, and downy layers of feathers. Briefly, for each feather layer, we calculated reflectance, transmission, and absorption spectra from 300 to 700 nm as a function of the pigments and nanostructures within each layer. Total reflectance for a given patch is the sum of three main paths that interact with the feather layers: (i) light that is reflected from the outermost colorful layer, (ii) light that is transmitted through the colorful layer and reflected by the achromatic layer back through the colorful layer, and (iii) light that is transmitted through both colorful and achromatic layers and reflected by the downy layer back through colorful and achromatic layers. Further details and equations—and a discussion of the model’s limitations—are given in the “Extended methods” section of the Supplementary Text.

We used our optical model to understand the effects of achromatic background reflectance and absorption on the brightness and saturation of pigmented and structurally colored plumage. We calculated total patch reflectance across a wide range of simulated carotenoid optical densities and structural color parameters on either white, gray, or black achromatic layers and gray downy layers. We used pavo to calculate the brightness, saturation, and hue of each simulated reflectance spectrum as described in the “Microspectrophotometry” section above. We also used avian visual modeling to explore the effects of achromatic background reflectance on brightness and saturation from the avian visual perspective. We used the vismodel() function from pavo to plot each of our simulated spectra in the avian tetrahedral color space (*46*) and calculated the relative quantum catch of each photoreceptor involved in color vision (ultraviolet-sensitive, short-wave-sensitive, medium-wave-sensitive, and long-wave-sensitive) and luminance perception (the double cone) using the blue tit (*Cyanistes caeruleus*) visual system and ocular media transmission. We assumed ideal illumination conditions (constant illumination across all wavelengths). We extracted measures of avian luminance (the relative stimulation of the avian double cone, a measure of brightness) and chroma (the distance from the achromatic center of the tetrahedron, a measure of saturation).

### Avian visual modeling of female and male reflectance spectra

We used avian visual modeling to estimate the perceptual distances between female and male color. As described above, we used pavo to model each spectrum in avian tetrahedral color space, but here, we calculated raw rather than relative quantum catches for each photoreceptor. We used the function coldist() in pavo to calculate the noise-weighted Euclidian chromatic (∆*S*) and luminance (∆*L*) distances between spectra according to the receptor noise-limited model (*49*).

We used this approach to quantify sexual dichromatism (the perceptual distance between female and male color as measured with reflectance spectrophotometry from specimens). We also used this approach to estimate the perceptual distance between pigmented female and male feather barbs measured with MSP for the three *Tangara* carotenoid patches for which females and males had black and white achromatic regions, respectively. We first calculated ∆*S* and ∆*L* on the same background (both sexes on black) and second on backgrounds corresponding to the color of the achromatic layer on each sex (females on black and males on white).

### Feather measurements

For each feather (*n* = 72), we measured the total feather length and length of the colorful, achromatic, and downy regions along the rachis from multispectral images in ImageJ (see the “Extended methods” section of the Supplementary Text for further details). We used these measurements to compare colorful and achromatic feather features between females and males.

### Imaging of achromatic layers in tanagers and passerines

We used a combination of confocal microscopy (a Keyence VK-X3050 at the Princeton University Imaging and Analysis Center and a Keyence VHX-5000 at the Natural History Museum of Los Angeles County), macro photography (Canon EOS R camera body and Canon EF 100mm f/22.L IS USM Macro Lens; Canon, Tokyo, Japan), and iPhone SE photography to image colorful and achromatic layers on whole specimens. For confocal images, we placed specimens on the microscope stage and captured images at a range of focal planes, which were stitched together using either VK-X3000 or VHX-5000 software (Keyence, Itasca, IL). We first imaged colorful plumage on specimens to understand the natural positioning of individual feathers. Next, we used pins to move the colorful tips of feathers and expose achromatic layers. Where applicable, we also imaged feathers at the edge of patches where there was a sharp transition between coloration mechanisms and documented associated switches in feather achromatic region color.

## Supplementary Materials

### This PDF file includes:

Supplementary Text Figs. S1 to S16 Tables S1 to S5 References
