# Supplementary material for: Hidden white and black feather layers enhance plumage coloration in tanagers and other songbirds
Source: Sci Adv. 2025 Jul 23;11(30):eadw5857. doi: 10.1126/sciadv.adw5857 (PMC12285719; doi:10.1126/sciadv.adw5857)
Supplement: Supplementary file 1 — Supplementary Text Figs. S1 to S16 Tables S1 to S5 References [file sciadv.adw5857_sm.pdf]

Science Advances
AAAS

# Supplementary Materials for

## Hidden white and black feather layers enhance plumage coloration in tanagers and other songbirds

Rosalyn M. Price-Waldman *et al.*

Corresponding author: Rosalyn M. Price-Waldman, rosalynp@princeton.edu

*Sci. Adv.* **11**, eadw5857 (2025)
DOI: 10.1126/sciadv.adw5857

**This PDF file includes:**

Supplementary Text
Figs. S1 to S16
Tables S1 to S5
References

## Supplementary Text

Extended results

***Variation in achromatic layers in colorful passerines***

We surveyed the color of hidden feather layers in passerine lineages with feathers colored by melanin pigments and compared them to lineages with carotenoid pigmentation and structural coloration. We identified white achromatic layers paired with carotenoid-pigmented plumage and black achromatic layers paired with structurally colored plumage in many colorful passerine lineages (see *Main text: Results*), but we also identified several intriguing exceptions.

First, we found dark, velvety carotenoid-pigmented feathers paired with black achromatic layers in silver-beaked tanagers (*Ramphocelus carbo*) and pale blue structurally colored feathers paired with white achromatic layers in superb fairywrens (*Malurus cyaneus*) and variegated fairywrens (*Malurus lamberti*) (table S5; fig. S15). These examples are consistent with evidence that achromatic layers have large impacts on the color of visible plumage but also suggest that these plumage patches may be under selection for dark carotenoid-pigmented reds and light, unsaturated structurally colored blue plumage. Further characterization of achromatic layers in colorful birds will likely reveal additional interesting optical effects that arise as a result of optical interactions among hidden and visible layers of plumage.

Second, we found that the prominence of achromatic regions varies across the body in some species. For example, in two iconic red birds – northern cardinals (*Cardinalis cardinalis*) and house finches (*Haemorhous mexicanus*) – achromatic regions are completely lacking in some carotenoid-pigmented plumage patches but present in others (table S5; fig. S16). In both these species, the crown feathers transition directly from a plumulaceous region to a pigmented pennaceous region (fig. S16). The lack of white achromatic layers in the crowns of these species is surprising because social and sexual selection likely act on plumage brightness in both species (*14*, *84*). Notably, both species occur in temperate areas, in contrast to many of the colorful tropical species (including *Tangara* tanagers) that we examined. In this case, selection for the thermoregulatory benefits of having a large plumulaceous feather region in colder climates may explain why these species lack a white achromatic region in some body regions, despite selection for conspicuous red plumage (*14*, *84*). In these patches, white light scattering from air spaces within the pigmented portion of the barb (in lieu of a white hidden layer) may be a crucial component of color production. Further characterization of hidden feather layers across latitudinal or other ecological gradients is likely to reveal informative patterns of variation in achromatic layers.

Extended methods

***Classifying Tangara patches by color production mechanism and selecting focal patches***

We examined six standard patches in male *Tangara* tanagers of 26 species. We classified each patch by color production mechanism according to several lines of evidence. We first examined the shape of the reflectance spectrum for each patch, as reflectance spectra for pigments and structural colors have characteristic shapes. We next examined the dorsal and ventral surfaces of the feather and cross-sections of individual

barbs using a Keyence VK-X3050 confocal microscope (Keyence Corporation, Osaka, Japan) at the Princeton University Imaging and Analysis Center. In some cases, we also used a Quanta 200 FEG Environmental-SEM (Thermo Fisher Scientific, Waltham, MA, USA) at the Princeton University Imaging and Analysis Center to image feather barb cross-sections and determine whether nanostructures were present within feather barbs. We classified red and yellow feathers as "carotenoid-pigmented" if the reflectance spectrum had a typical carotenoid shape, the obverse and reverse surfaces looked similar and showed no evidence of structural color, and the associated cross sections of the barb ramus showed no evidence of a quasi-ordered nanostructure characteristic of non-iridescent structural color. We classified feathers as "melanin-pigmented" using similar reasoning. We classified feathers as "structurally colored" if the reflectance spectrum had a typical structural shape, if the obverse and reverse surfaces were differently colored or had clearly visible medullary cells, and if the cross-sections showed a quasi-ordered nanostructure the reflected blue or violet light but no evidence of carotenoids or other pigments in the cortex. We classified feathers as "structure-pigment" if we observed both carotenoids and nanostructures. We recorded the color of the achromatic feather regions for each of these patches. In general, we examined a single specimen from each species. However, when sampling feathers from species where the achromatic layers were different between females and males (*i.e.*, black in females and white in males), we examined several individuals of each sex to ensure that the observed color of the achromatic layer was consistently different between sexes.

We chose to focus on feathers that were carotenoid-pigmented or structurally colored but not both to simplify the interpretation of our experimental results, because the effects of achromatic layer reflectance on feathers with both pigments and nanostructures are likely to be complicated. There was one exception: in the case of the *Tangara chilensis* specimen where we removed colorful feather tips to expose achromatic layers, we chose to remove the feather tips from the red rump and green crown. The rump is colored by carotenoid pigments, while the crown contains both nanostructures and carotenoid pigments that interact to produce green. In this case, we selected the *T. chilensis* specimen rather than another specimen because this specimen was from an aviary and was an appropriate candidate for destructive sampling of feathers. We observed that green feather layers tend to be paired with black achromatic layers, like most structurally colored plumage, and so we reasoned that characterizing the black achromatic layer in *T. chilensis* crown was comparable to characterizing the black achromatic layer in a blue structurally colored patch. We chose to remove feathers from the green crown rather than the blue structurally colored breast to match the sampling of crown feathers from the rest of the specimens.

***Reflectance of Calibrite Color backgrounds***

We used the white and black squares from a Calibrite 3-step GrayScale Color Checker photography standard (Calibrite LLC, Wilmington, DE, USA) as the background in our microspectrophotometry (MSP) measurements, and the black and second-brightest white squares from a Calibrite ColorChecker Classic photography standard as the backgrounds in our multispectral images. We measured the reflectance of these backgrounds using spectrophotometry (see main text for spectrophotometry methods) and compared these measurements to reflectance spectra of exposed white and

black achromatic layers in the *Tangara chilensis* specimen to ensure that our achromatic backgrounds had biologically realistic reflectance (fig. S1).

***Defining colorful, achromatic, and downy regions of feathers***

We developed a set of criteria to define boundaries between downy, achromatic, and colorful feather regions. Microscopic examination of the colorful regions in the pennaceous part of the feather in tanagers revealed a number of specializations for color production, including wide barb rami that lack barbules. We defined the boundary between the achromatic and colorful regions as the place where barbs become visibly wider and transition from either black or white to blue or yellow/red. Changes in barb width were usually coincident with reduction in barbules, except in belly feathers.

Downy (plumulaceous) barbules in passerines have characteristic morphologies, including slightly widened, rounded nodes (junctions between adjacent barbule cells) and transparent villi at the base of the barbule cells, with melanin granules restricted to the nodes (*85*). We defined the boundary between the downy and achromatic regions as the place where barbules transition from downy morphologies with melanin restricted to nodes to either lacking melanin completely (white achromatic regions) or being fully melanized (black achromatic regions). In most cases, the change in melanin deposition also coincided with changes in barbule morphology from plumulaceous to pennaceous, but in rare cases (for example, *Tangara chilensis* rump; Fig. 1Cd) the white achromatic region also included plumulaceous barbules (long and fluffy, with expanded nodes). In these cases, we considered the achromatic region to be the area lacking melanin (*i.e.*, the white region).

We used the criteria above to define colorful, achromatic, and downy regions in our multispectral images, and restricted our measurements of HSL values to each region as described in *Materials and Methods: Multispectral Imaging*.

***Feather length measurements***

All feather measurements were made on multispectral images of individual feathers on white backgrounds. We measured the size of downy, achromatic, and colorful feather regions along the length of the rachis (the central shaft of the feather) using the line segment tool in ImageJ v. 1.53 (*80*). We measured total feather length from the base of the rachis (excluding the calamus) to the tip of the most central colorful barb. We measured the length of the achromatic and colorful regions following the criteria described previously (see "*Defining colorful, achromatic, and downy regions of feathers*" above). We subtracted the lengths of the colorful and achromatic regions from the total feather length to give the length of the downy regions, and we also calculated the proportion of each feather occupied by each region. Each feather was measured twice by separate authors, and measurements for each feather were averaged after examining raw measurements to ensure there were no large discrepancies.

***Measuring light transmission through stacks of colorful and achromatic feather regions***

We used microspectrophotometry to measure the transmission of light through representative colorful and achromatic feather layers and used these values to guide parameter choice for our optical model. Using a CRAIC UV-Vis microspectrophotometer

(Chapel Hill Nanofabrication and Analytical Laboratory, Chapel Hill, NC), we collected transmission data from 350 – 700 nm using a 40x objective and took 60 scans to average. We collected a dark standard by taking a scan without the light source and a light standard by measuring transmission through a blank slide. To measure the transmission of light through white achromatic layers, we placed three *Tangara chilensis* male rump feathers on a slide so that the white achromatic regions were stacked. We examined achromatic layers on specimens and found that achromatic layers generally vary between one and three feathers in depth, depending on the body region. We reasoned that measuring three stacked feathers would provide a conservative estimate for transmission, with the caveat that the actual transmission of the achromatic layer likely varies in different body regions. We lightly taped feather tips so that feathers were touching, similar to their arrangement on the body of a bird. We took readings at twelve points across the stacked achromatic regions, including six points through barbs and six points through barbules. To measure the transmission of light through black achromatic layers, we repeated the same process on three stacked *T. mexicana* rump feathers. Finally, we measured the transmission of light through structurally colored feather layers. We stacked three *T. mexicana* rump feathers and measured transmission across nine barbs. Using pavo, we smoothed transmission spectra using a span of 0.3, corrected negative values by setting them to 0, and averaged the repeated spectra.

We used measurements of transmission through white and black achromatic layers and blue structurally colored layers to estimate reasonable values for the achromatic layer absorption and the absorption of the basal melanin layer in structurally colored barbs, respectively (see *Optical model* below for more details). Specifically, we compared our transmission values to reflectance spectra and estimated reasonable values for absorption, assuming that all light is either reflected, transmitted, or absorbed. However, transmission measurements are not directly comparable to reflectance measurements from the MSP. While both approaches measure the percentage of light reflected or transmitted at each wavelength of light, transmission is measured absolutely in terms of the intensity of photons that passes through a sample (feathers stacked on a slide) relative to a blank slide, and reflectance is measured relative to a standard (in this case, a 99% reflective Spectralon standard; Labsphere, North Sutton, NH, USA, see *Materials and Methods*). We therefore estimated reasonable, but approximate, absorption values (see *Optical model* below).

***Optical model***

We modeled total plumage reflectance accounting for optical interactions between colorful, achromatic, and downy feather layers. Each layer reflects, absorbs, and transmits some fraction of light at each wavelength ($\lambda$) from 300 – 700nm. Following a previous optical model of the dermal chromatophore unit (*43*), we approximated total plumage reflectance as the sum of the three primary light paths:

1) $R_{Colorful}(\lambda)$, light reflected from colorful layer

2) $T_{Colorful}(\lambda)^2 R_{Achromatic}(\lambda)$, light reflected from the achromatic layer that passes twice through the colorful layer

3) $T_{Colorful}(\lambda)^2 T_{Achromatic}(\lambda)^2 R_{Downy}(\lambda)$, light reflected from the downy layer that passes twice through the achromatic layer and twice through the colorful layer

We assumed that any light transmitted through the downy layer would be absorbed by the skin. Below, we provide details of the model for each layer.

**Colorful layer: Carotenoids**

The reflectance of carotenoid-containing plumage depends on the selective absorption of light by carotenoid pigments within the feather and light scattering from keratin (*27*). We therefore calculated reflectance, absorption, and transmission spectra for both carotenoids and keratin within this layer and calculated the total reflectance of this layer as the product of keratin reflectance and carotenoid transmission.

To calculate carotenoid absorption and transmission, we used a normalized absorbance curve from astaxanthin, a ketocarotenoid that produces red coloration in many birds (*86*). A normalized astaxanthin curve obtained by High-Performance Liquid Chromatography was provided by Jocelyn Hudon. We assumed that reflectance from the carotenoid pigments themselves is negligible (*27*) and that light is either absorbed by carotenoids or transmitted. We calculated transmission through carotenoids as

$$T_{Carotenoid}(\lambda) = 10^{-cA(\lambda)}$$

where $c$ is the optical density of the carotenoid layer at the wavelength of peak absorption and $A(\lambda)$ is the absorbance spectrum of astaxanthin. Optical density is directly proportional to both pigment concentration and the path length of light through a pigmented layer (*42*), so variation in simulated optical density is a proxy for changes in pigment concentration or the thickness of the colorful layer. Our approach is very similar to approaches which have previously been used to calculate transmission of light through carotenoid-pigmented oil droplets in the retina (*87*) and reflectance of light from carotenoid-pigmented feathers (*88*). Note that other authors using the above equation to simulate the reflectance of a carotenoid-pigmented feather (*88*) assume that all light transmitted through pigments is then reflected back to the surface of the feather by an unspecified, uniformly reflecting background, so that transmission is equal to reflectance; we do not make the same assumption here.

We approximated the reflectance of unpigmented keratin using a reflectance spectrum of the exposed white achromatic layer in *T. chilensis* (fig. S1). Because the reflectance of the white achromatic layer may be different than the reflectance of unpigmented keratin within the colorful region of a feather, we rescaled the reflectance spectrum to a maximum reflectance of 40% based on a study which experimentally removed carotenoid pigments from a formerly yellow feather and measured the reflectance of the resulting unpigmented keratin (*27*). Consistent with other optical models of avian feathers, we assumed that absorption by keratin within the carotenoid-keratin layer would be inconsequential (*89*). We checked the validity of this assumption by conducting a set of simulations that included a simulated keratin absorbance curve and found that overall results were similar, so in our final simulations we assumed no absorption by keratin. We therefore calculated light that is transmitted through keratin as

$$T_{Keratin}(\lambda) = 1 - R_{Keratin}(\lambda)$$

Finally, we calculated the reflectance of the colorful carotenoid-keratin layer at each wavelength as

$$R_{Colorful}(\lambda) = T_{Carotenoid}(\lambda) \times R_{Keratin}(\lambda)$$

and the transmission of the carotenoid-keratin layer as

$$T_{Colorful}(\lambda) = T_{Carotenoid}(\lambda) \times T_{Keratin}(\lambda)$$

**Colorful layer: structural colors**

Following a model of multilayer color production in the dermal chromatophore unit (*43*) (see above), we modeled the structurally colored layer of plumage by using an empirical structural reflectance spectrum and applying two transformations that simulate changes in the underlying nanostructures and microstructures. Applying transformations to an empirical reflectance spectrum is a simplified approach that allowed us to generate biologically relevant variation in reflectance spectra without directly modeling the complex nanostructures and microstructures that produce structural coloration. We used the reflectance spectrum of a blue *Tangara velia* male epaulet, which we measured from a male specimen (LACM 28102) following the spectrophotometry approach described in *Materials and Methods*.

Reflectance from the structural layer is

$$R_{Structural}(\lambda) = r(\mathrm{R}(\lambda) + v)/\max(\mathrm{R}(\lambda) + v))$$

where $R(\lambda)$ is the empirical reflectance spectrum and $r$ and $v$ are reflectivity and dilution transformations, respectively (*43*). Changes in $r$ alter the brightness of the spectrum produced, while changes in $v$ alter the shape of the spectrum by ‘diluting’ the short-wavelength spectral peak with reflectance from longer wavelengths (essentially shifting the color away from a saturated blue towards white). While changes in $r$ and $v$ do not directly simulate changes in specific nanostructural features, empirical work on non-iridescent structural colors in feathers have linked changes in brightness and spectral saturation to the number and regularity of the keratin rods and the size of internal air spaces in the nanostructure, as well as the thickness of the keratin cortex and microstructural features including barb size and barbule density (*41*, *90*, *91*). Reflectivity and dilutions transformations are therefore proxies for biologically relevant variation in nanostructures, including thicker colorful layers. For simulations where we varied $r$ we set $v$ to 0, and for simulations where we varied $v$ we set $r$ to 0.7 (the reflectance value corresponding to the original *T. velia* reflectance spectrum).

We assumed no absorption of light by the keratin nanostructure in the structurally colored layer (*89*). However, a key feature of structural colors in feather barbs is a layer of melanin located beneath the nanostructure within the barb ramus, generally referred to as the basal melanin layer (*20*, *35*). The basal melanin should absorb some fraction of the light which is not reflected by the nanostructure; therefore, we modeled the effects of basal melanin on the structural layer by including an absorption fraction which represents broadband absorption of light. We set the absorption fraction to 0.25 for all simulations.

This value is arbitrary, but we determined it to be reasonable based on measurements of light transmission through the blue colorful regions of stacked structurally colored feathers (mean transmission 9.4%) that we collected using MSP (see *Measuring light transmission through stacks of colorful and achromatic feather regions*). We calculated the absorption of the structural layer as

$$A_{Structural}(\lambda) = \left(1 - R_{Structural}(\lambda)\right) \times 0.25$$

Transmission of light through the structural layer is

$$T_{Structural}(\lambda) = 1 - R_{Structural}(\lambda) - A_{Structural}(\lambda)$$

**Achromatic layers**

We modeled white, gray, and black achromatic layers as spectrally flat, and varied reflectance and absorption. Based on hyperspectral measurements of the exposed achromatic layers on *Tangara chilensis* (Fig. 1D), we set the reflectance of white achromatic layers to be 55% and the reflectance of black achromatic layers to be 5%. We modeled gray achromatic layers with a reflectance of 30%, intermediate between white and black achromatic layers. We set the absorption of white achromatic layers to be 30%, the absorption of black achromatic layers to be 90%, and the absorption of gray achromatic layers to be 60%. Transmission is therefore 15% for white achromatic layers, 5% for black achromatic layers, and 10% for gray achromatic layers. These values are similar to our empirical measurements of light transmission through white achromatic layers (12.2%) and black achromatic layers (2.1%).

**Downy layers**

We modeled the downy layer as a spectrally flat 'gray' layer with 30% reflectance and 60% absorption.

**Simulations**

To predict the effects of achromatic layer reflectance on the brightness and saturation of colorful plumage, we simulated carotenoid-pigmented and structurally colored spectra on white, gray, and black achromatic layers and gray downy layers. We varied carotenoid optical density ($c$) from 0 to 10, structural color reflectivity ($r$) from 0 to 1, and structural color dilution ($v$) from 0 to 1. We kept all other parameters for white, gray, and black achromatic layers and downy layers constant. In total, we modeled the reflectance of 99 plumage patches (11 carotenoid optical densities, 11 structural colors with varying reflectivity transformations, and 11 structural colors with varying dilution transformations over white, gray, and black achromatic layers).

**Limitations of optical modeling**

We note that the optical model presented here and extended from a previous model of the dermal chromatophore unit (*43*) contains a set of simplifying assumptions with a number of associated caveats. We expand on these limitations below and suggest avenues for future research.

In our model, the total reflectance of the outer layer of carotenoid-pigmented feathers depends on light absorption by carotenoids and light scattering by the feather

keratin in which carotenoids are embedded. Following earlier approaches (*87*, *88*), we used a normalized carotenoid absorbance curve and varied carotenoid optical density to calculate transmission spectra for carotenoids. We then multiplied our simulated carotenoid transmission spectra by an unpigmented keratin reflectance spectrum to estimate the overall reflectance spectrum of the outer layer of colorful carotenoid-pigmented feathers. Our empirical unpigmented keratin reflectance spectrum and the associated simulated carotenoid reflectance spectra (fig. S2) resemble the empirical reflectance spectra of a carotenoid-pigmented feather before and after carotenoid extraction (*27*). However, we note that multiplying carotenoid transmission by keratin reflectance does not account for the complex geometry of carotenoid pigments embedded in keratin within feather barbs. At present, the internal architecture of carotenoid-containing barbs has not been described for most avian species; however, modeling the interplay of keratin scattering and carotenoid absorption would generate more accurate simulations of carotenoid spectra.

Similarly, we followed the approach presented in (*43*) and simulated the reflectance of the outer layer of structurally colored feathers by applying transformations to an empirical reflectance spectrum rather than directly modeling light reflectance from the quasi-ordered keratin-air nanostructures that produce structural coloration in the barb rami. We included an absorption fraction to account for the broadband absorption of light by a basal melanin layer within each structurally colored barb ramus. Modeling light scattering from nanostructures while accounting for the shape of the barb ramus and light absorption by melanin would enhance the accuracy of simulated structurally colored spectra. A useful approach for modeling complex optical interactions between light scattering and absorption within both carotenoid-pigmented and structurally colored barbs is Finite-difference Time-domain modeling, reviewed in (*92*).

**Supplementary Figures and Tables**

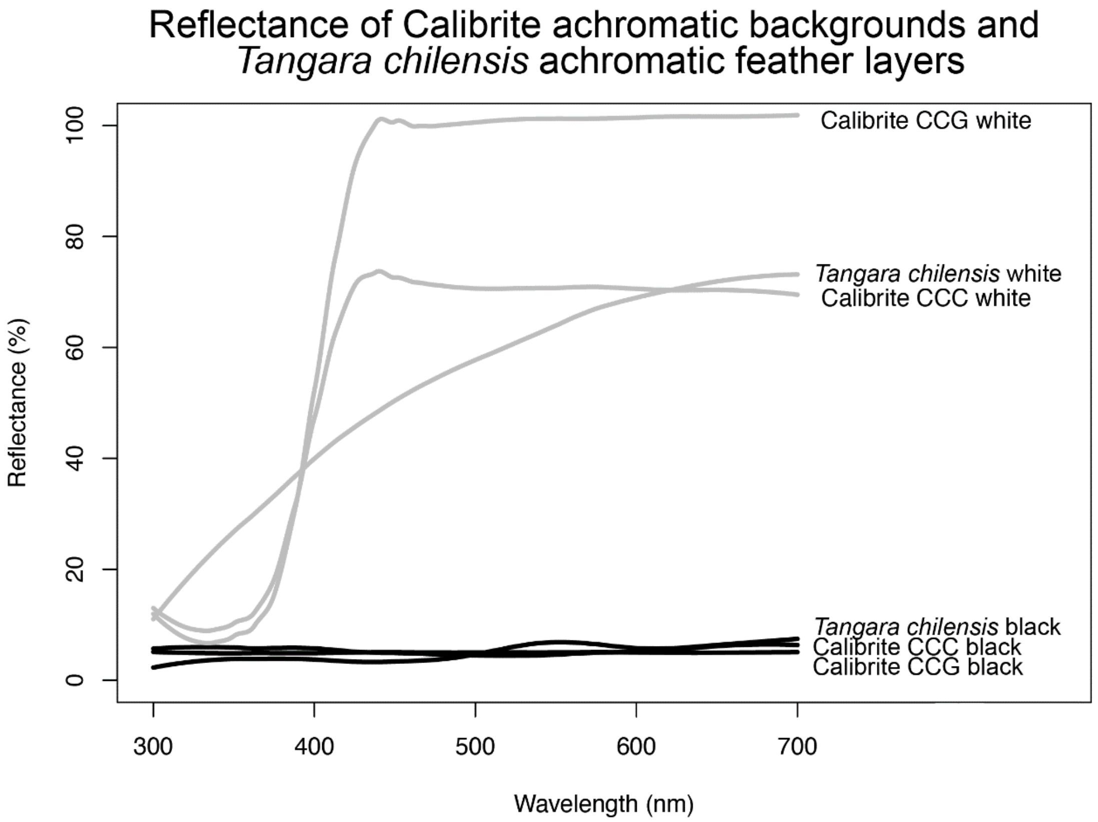


**Figure S1**. **Reflectance of white and black synthetic Calibrite backgrounds compared to white and black achromatic feather layers on a *Tangara chilensis* specimen.** We used the second-brightest white and darkest black squares on a Calibrite ColorChecker Classic (CCC) as the backgrounds in MP experiments and the white and black squares on a Calibrite ColorChecker 3-Step Grayscale (CCG) as the backgrounds in MSP experiments.

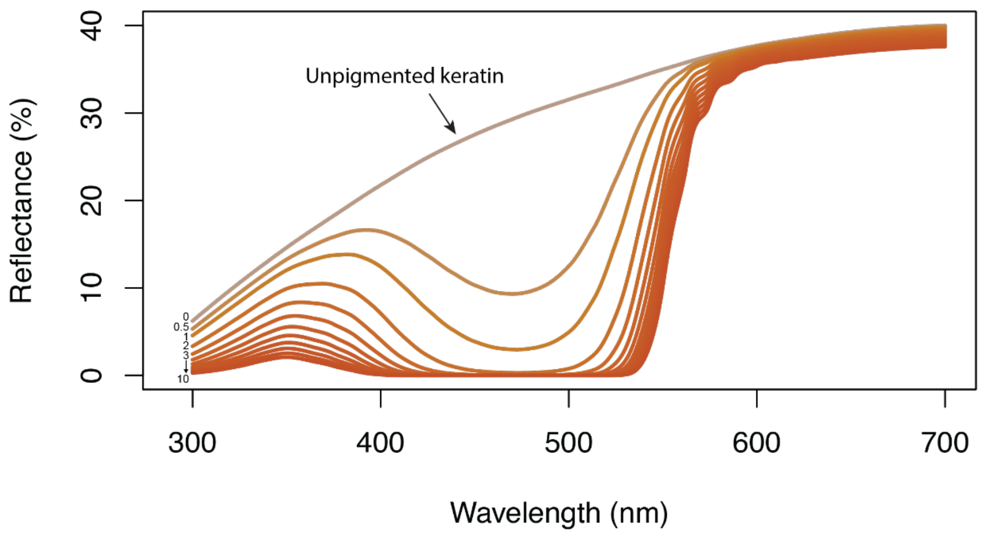


**Figure S2**. **Simulated reflectance from carotenoid-keratin layer.** We varied astaxanthin optical density ($c$) from 0 to 10. When the optical density is 0, the curve shows the reflectance of unpigmented keratin. The spectra above show the reflectance of the colorful carotenoid-keratin layer without accounting for optical interactions from achromatic or downy layers; we assume no reflectance from other feather layers. The spectra are colored according to their approximate human-perceived color, which we estimated using the pavo function spec2rbg().

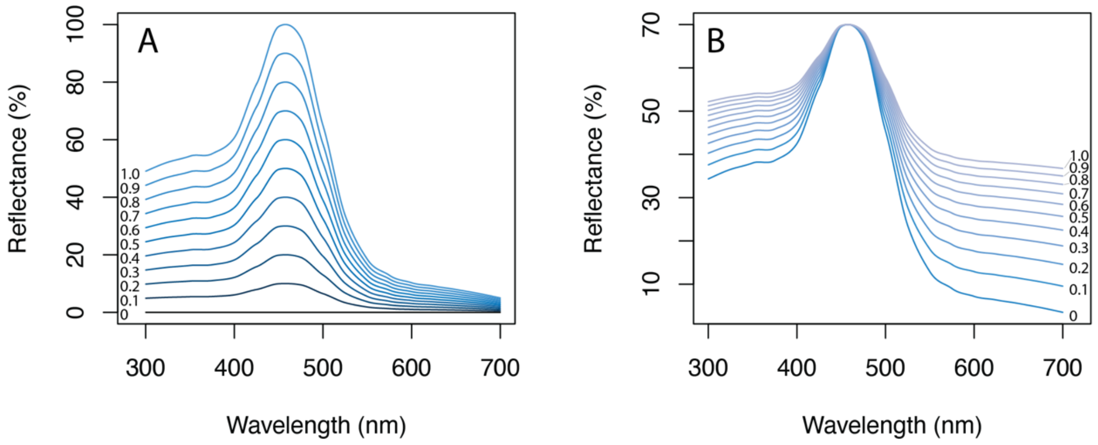


**Figure S3**. **Simulated reflectance from the structural layer.** We varied reflectivity ($r$) from 0 to 1 (A) and dilution ($v$) from 0 to 1 (B). The absorption fraction of basal melanin is set to 0.25 for all simulated spectra. The spectra above show the reflectance of the colorful structural layer without accounting for optical interactions from achromatic or downy layers; we assume no reflectance from other feather layers. The spectra are colored according to their approximate human-perceived color, which we estimated using the pavo function spec2rbg().

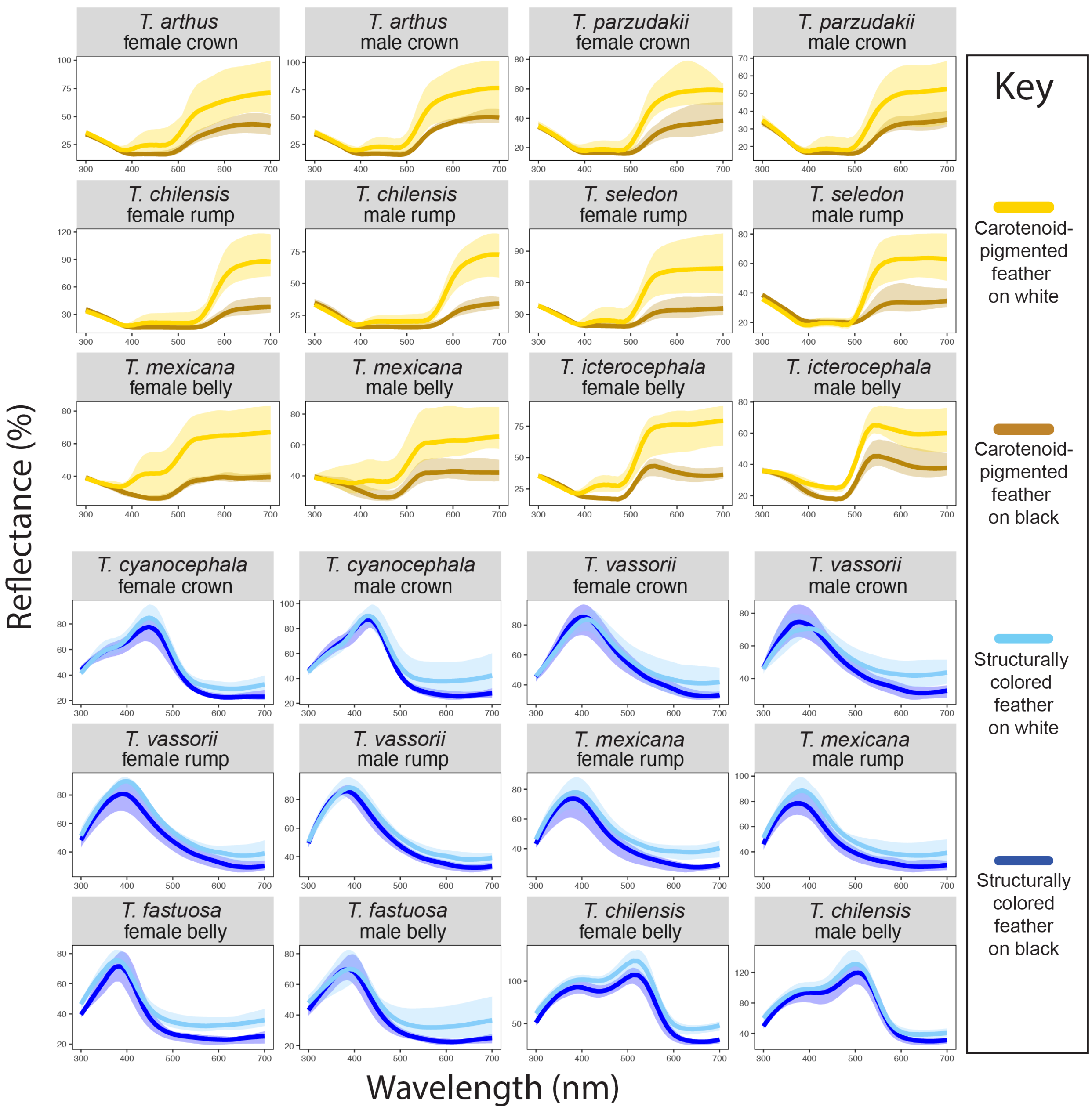


**Figure S4. Reflectance spectra of *Tangara* carotenoid-pigmented and structurally colored feathers on white and black achromatic backgrounds (Calibrite 3-Step Grayscale) measured with MSP.** Shaded regions correspond to the nine barbs measured for each feather, and solid lines correspond to the mean reflectance. White achromatic backgrounds increase the brightness of carotenoid-pigmented feathers and black achromatic backgrounds increase the saturation and decrease the brightness of structurally colored feathers.

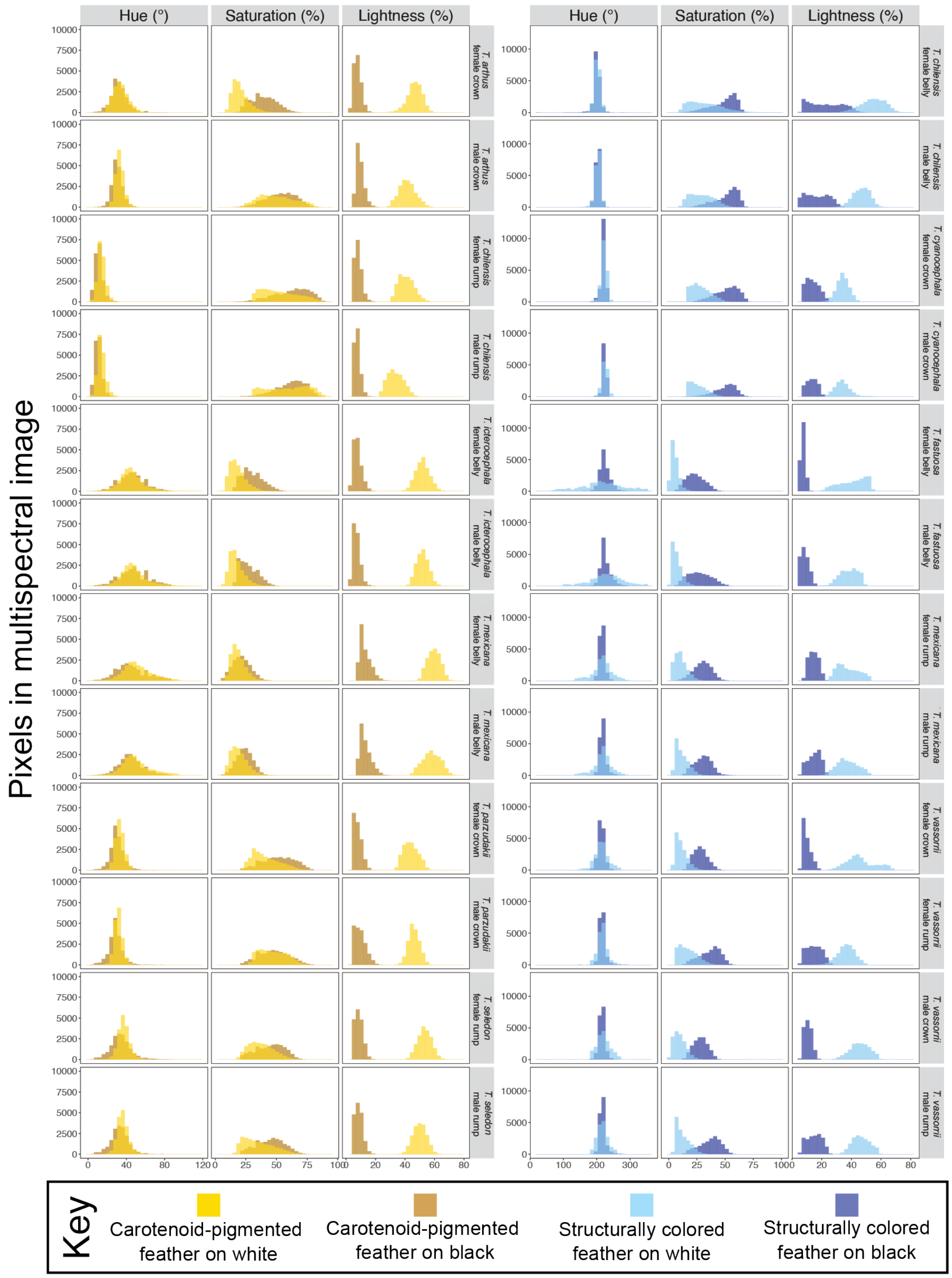


**Figure S5. Hue, saturation, and lightness of *Tangara* carotenoid-pigmented and structurally colored feathers on white and black achromatic backgrounds (Calibrite ColorChecker Classic) measured with MP.** White achromatic backgrounds increase the lightness of carotenoid-pigmented feathers and black achromatic backgrounds increase the saturation and decrease the lightness of structurally colored feathers, but achromatic backgrounds do not alter hue.

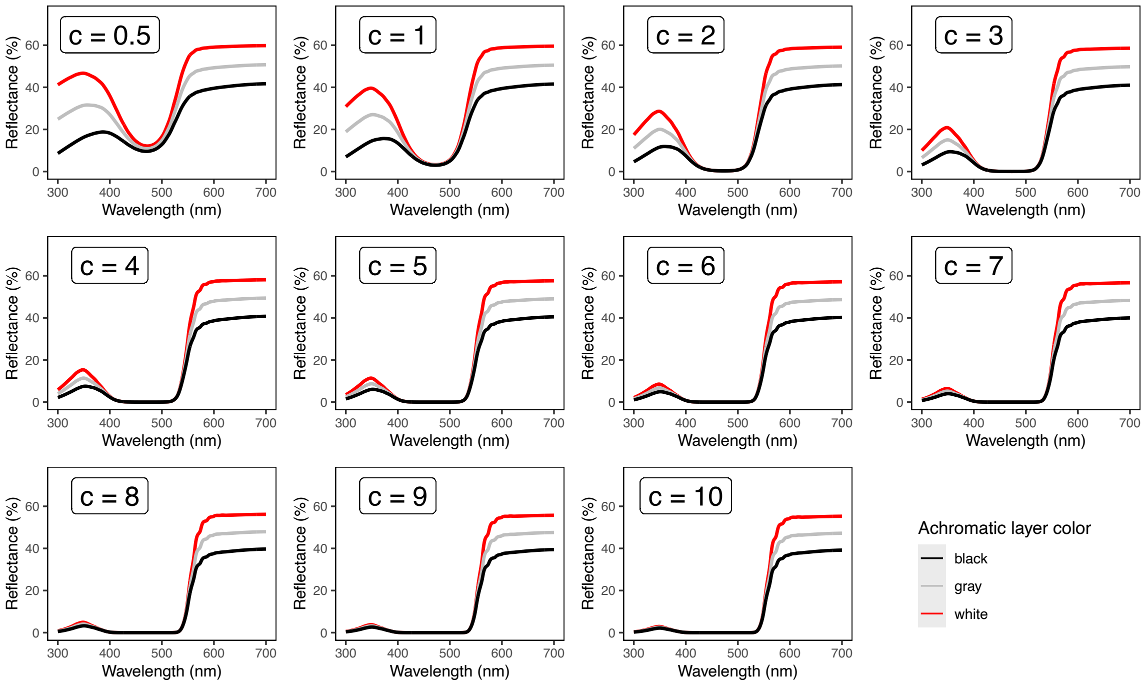


**Figure S6**. **Simulated carotenoid spectra on white, gray, and black achromatic backgrounds.** We used a normalized absorbance curve for astaxanthin (a ketocarotenoid which produces red colors in many birds) and varied the optical density (*c*) of carotenoid pigment from 0.5 – 10. For all simulations, we set the reflectance of white achromatic layers to 55%, the reflectance of black achromatic layers to 5%, and the reflectance of gray and downy layers to 30%. Across a wide range of simulated carotenoid optical densities, increasing reflectance from achromatic layers increases the total reflectance, resulting in brighter colors. As carotenoid optical density increases, reflectance from the achromatic layer has less of an effect on lower wavelengths (300 – 400 nm).

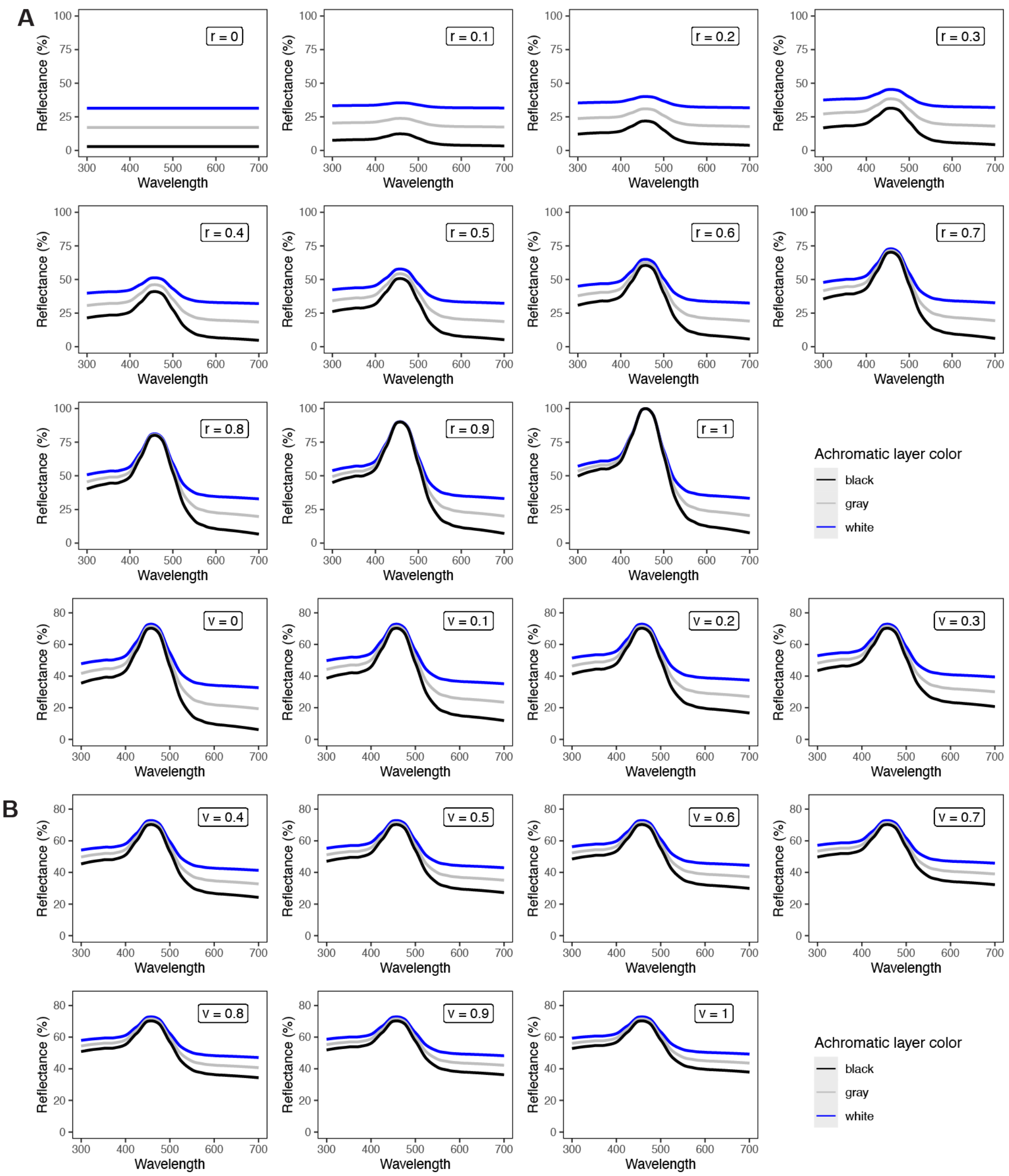


**Figure S7. Simulated structurally colored spectra on white, gray, and black achromatic backgrounds.** We used the reflectance spectrum of a structurally colored patch (*Tangara velia* epaulet) and applied a series of transformations (*43*) to the spectrum (reflectivity factor, *r*; dilution factor, *v*) to simulate changes in nanostructures. We varied *r* from 0-1 (A) and *v* from 0-1 (B) and set the absorption fraction of basal melanin within the barbs to 0.25. For all simulations, we set the reflectance of white achromatic layers to 55%, the reflectance of black achromatic layers to 5%, and the reflectance of gray and downy layers to 30%. Across both sets of transformations, increasing reflectance from the achromatic layers dilutes the structurally colored peak, resulting in a less saturated, brighter color.

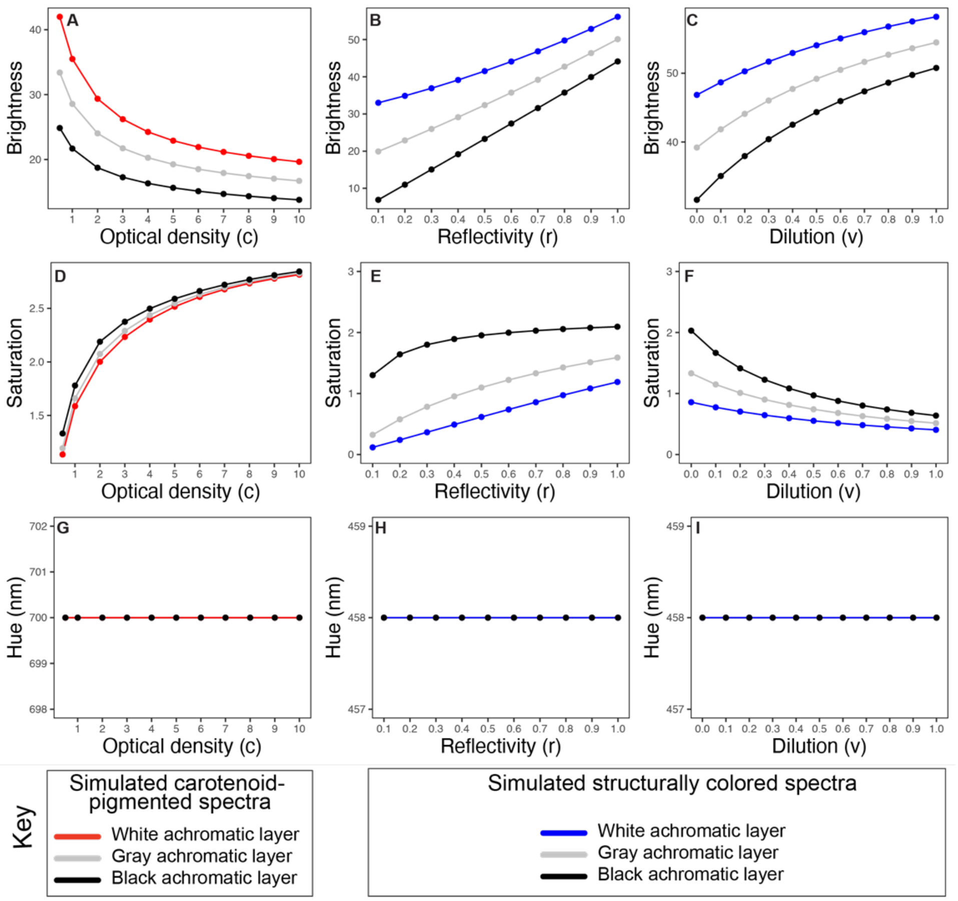


**Figure S8**. **Brightness, saturation, and hue measurements from simulated carotenoid-pigmented and structurally colored spectra on white, gray, and black achromatic layers across a range of carotenoid optical densities and structural color reflectivity and dilution factors.** Simulated carotenoid-pigmented spectra are shown in (A, D, G) and simulated structurally colored spectra are shown in (B-C, E-F, H-I). Brightness is B2 (the mean relative reflectance), saturation is S8 (($R_{max}$ – $R_{min}$)/B2), and hue is H1 (the wavelength of maximum reflectance, $R_{max}$). We calculated color summaries for each of the simulated spectra in fig. S6-S7 except structurally colored spectra where $r = 0$ (a flat spectrum, which cannot have hue or saturation). Achromatic layer reflectance primarily affects the brightness of carotenoid-pigmented spectra and the brightness and saturation of structurally colored spectra. Changes in brightness are consistent across a range of simulated carotenoid optical densities. In contrast, the achromatic layer has the greatest effect on structurally colored plumage when reflectance from nanostructures is low or when saturation is high. For highly reflective or unsaturated structural colors, achromatic layer reflectance has less pronounced effects either because most light is reflected directly from the colorful layer and not transmitted to the underlying achromatic layer, or because the spectrum is already so unsaturated that further backscattering of light from the achromatic layers will have relatively small further effects.

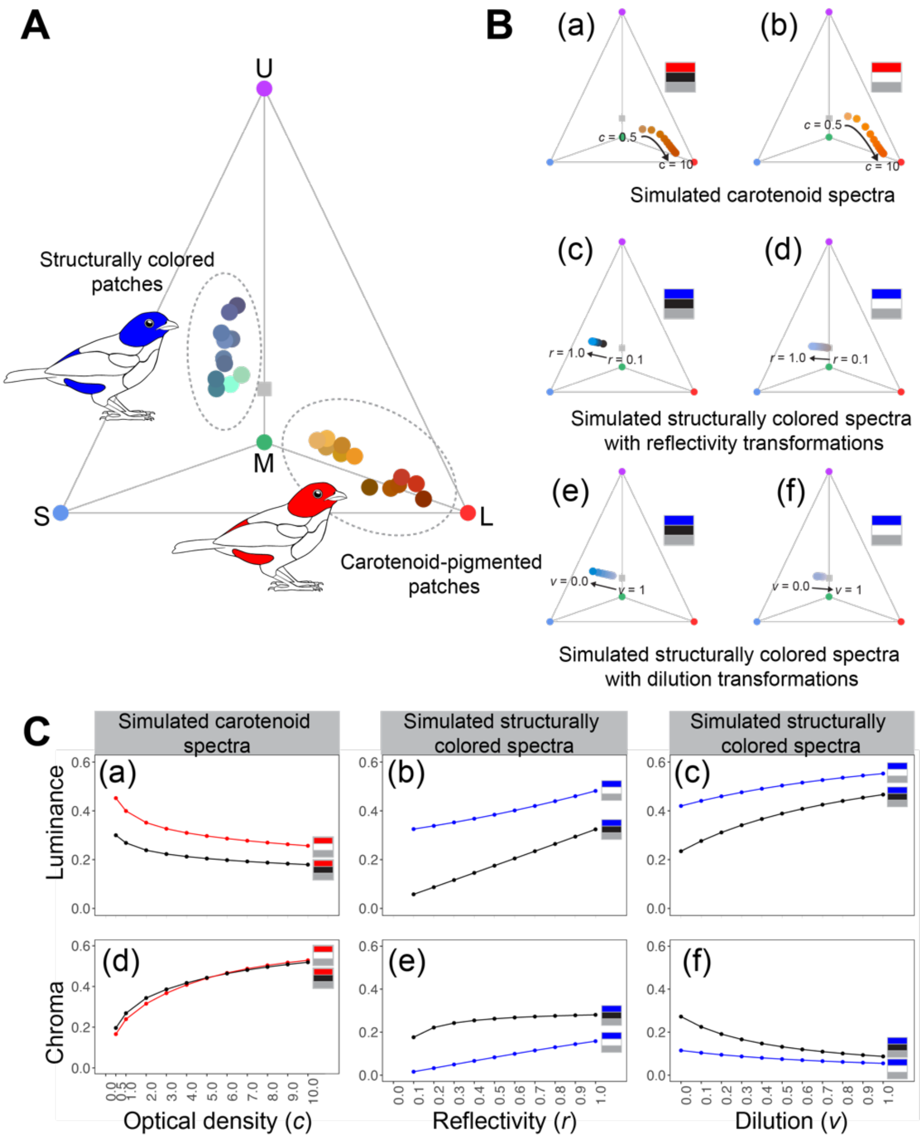


**Figure S9**. **Avian visual modeling of *Tangara* reflectance spectra and simulated carotenoid-pigmented and structurally colored spectra.** (A) shows the reflectance spectra of all carotenoid-pigmented and structurally colored patches that we sampled from specimens plotted in avian tetrahedral color space. The four vertices represent the four avian color cones (U, S, M, L) and the gray square represents the achromatic center of the tetrahedron (see text for details). (B) shows simulated carotenoid-pigmented (a-b) and structurally colored (c-f) spectra plotted in avian tetrahedral color space, separated by simulated achromatic layer color. Simulated carotenoid spectra (Ba-b) and simulated structurally colored spectra (Bc-f) occupy similar regions of color space as empirical measurements of plumage in (A). In (C), we show how luminance (a measure of brightness) and chroma (a measure of saturation) calculated from visual models of the simulated spectra change based on simulated variation in carotenoid optical density (a,d) and reflectivity and dilution transformations (b-c, e-f).

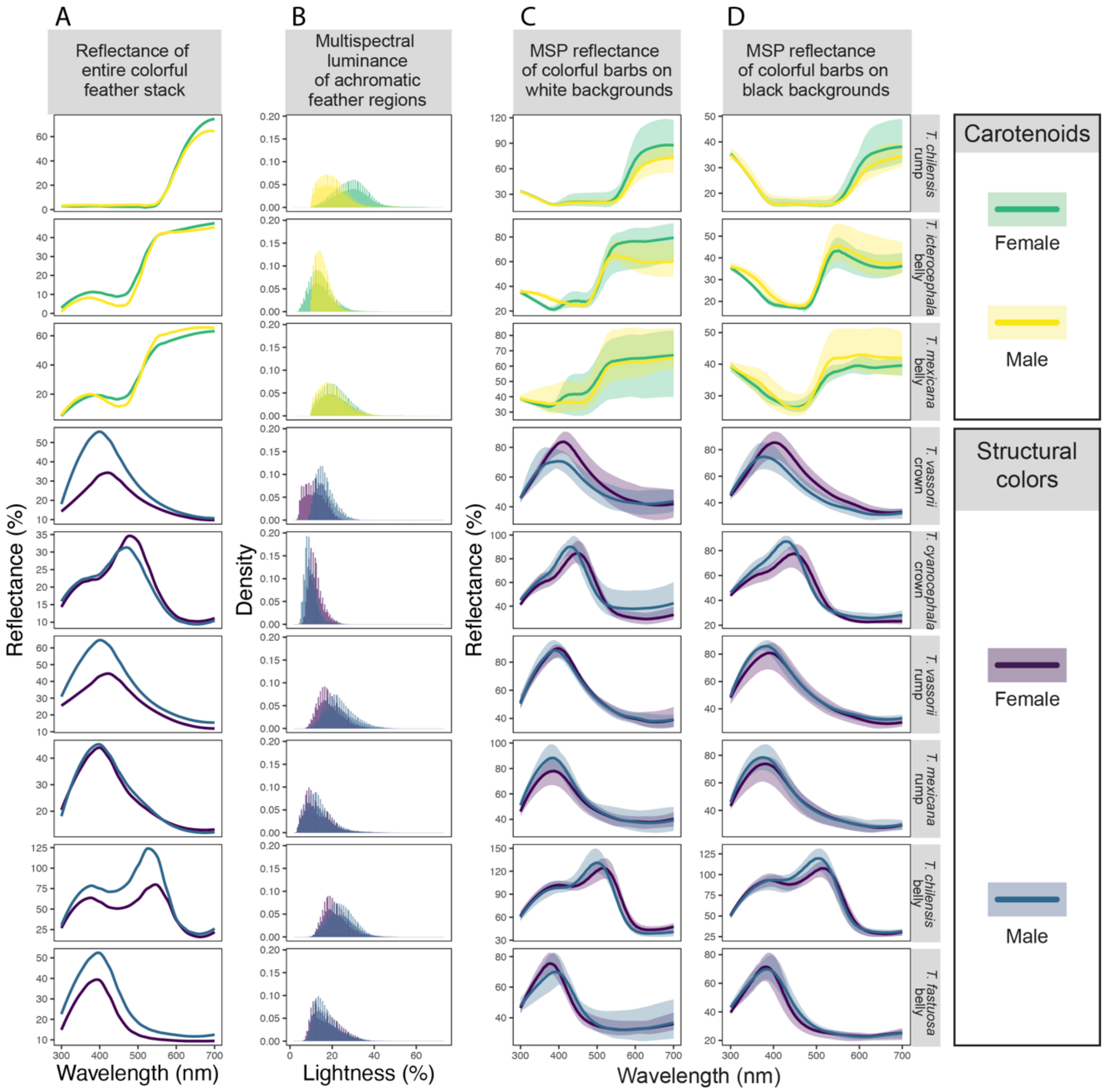


**Figure S10. Comparisons between female and male color for carotenoid patches not shown in Fig. 4 and structurally colored patches.** (A) shows the reflectance spectra for female and male focal patches measured on specimens, (C) shows achromatic feather region lightness measured with MP, (C) shows colorful feather region reflectance measured with MSP on white backgrounds, and (D) shows colorful feather region reflectance measured with MSP on black backgrounds. Reflectance in (A) is the average of three measurements from each patch taken with a spectrophotometer. To obtain the multispectral measurements of lightness in (B), we imaged white achromatic regions from pigmented feathers on black backgrounds and black achromatic regions from structurally colored feathers on white backgrounds. Shaded regions in C-D correspond to reflectance of nine barbs measured with MSP, and solid lines are the mean reflectance.

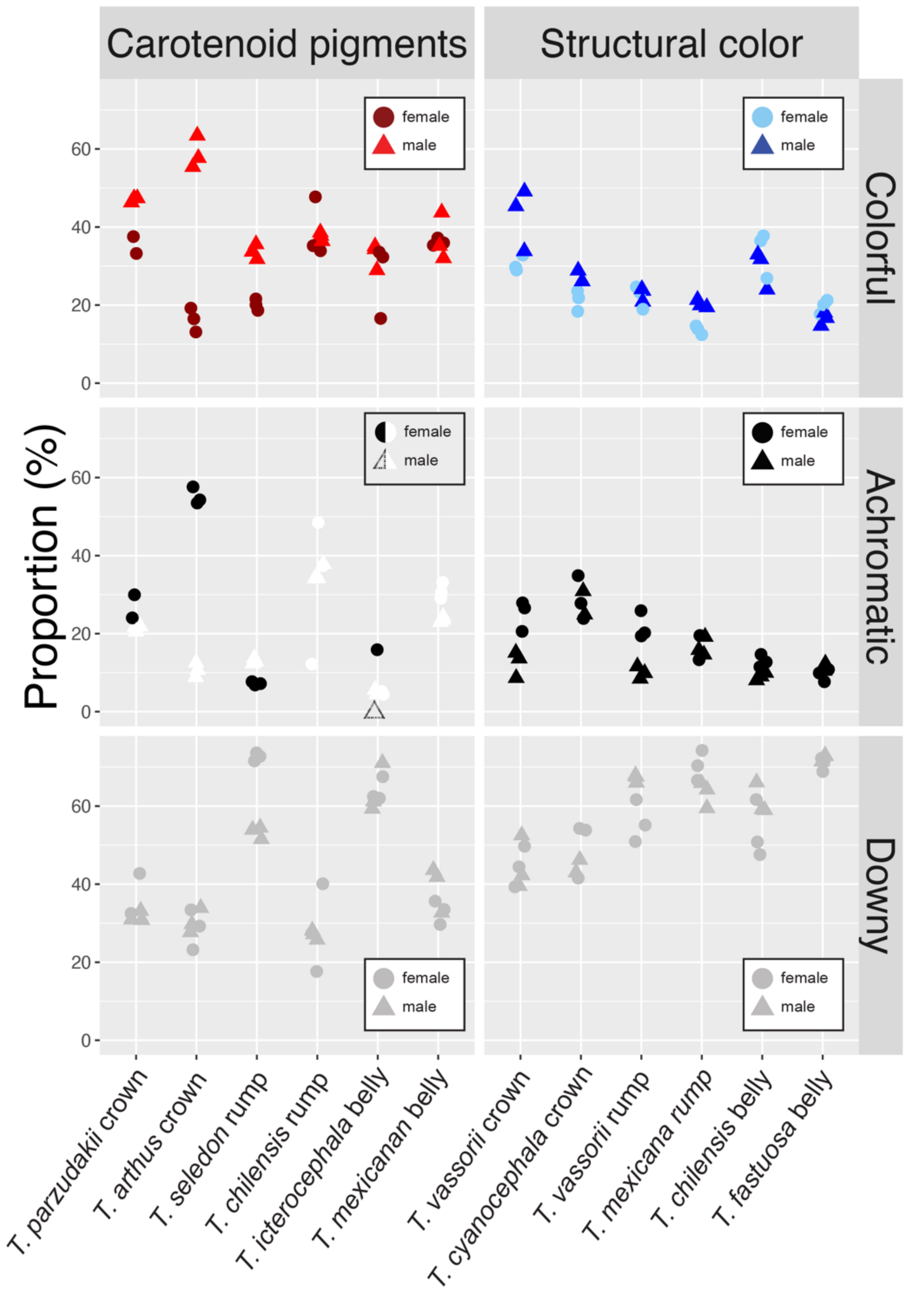


**Figure S11**. **Proportion (% of length) of colorful, achromatic, and downy regions of each *Tangara* feather in our dataset by coloration mechanism and sex.** Each of the three feather replicates for each patch and sex was measured independently by two authors. Achromatic regions are colored according to their actual color (white versus black), including the three carotenoid-pigmented patches where females had black rather than white achromatic layers. The triangle outlined in a dashed line indicates the measurement from a male *T. icterocephala* feather that lacked an achromatic region.

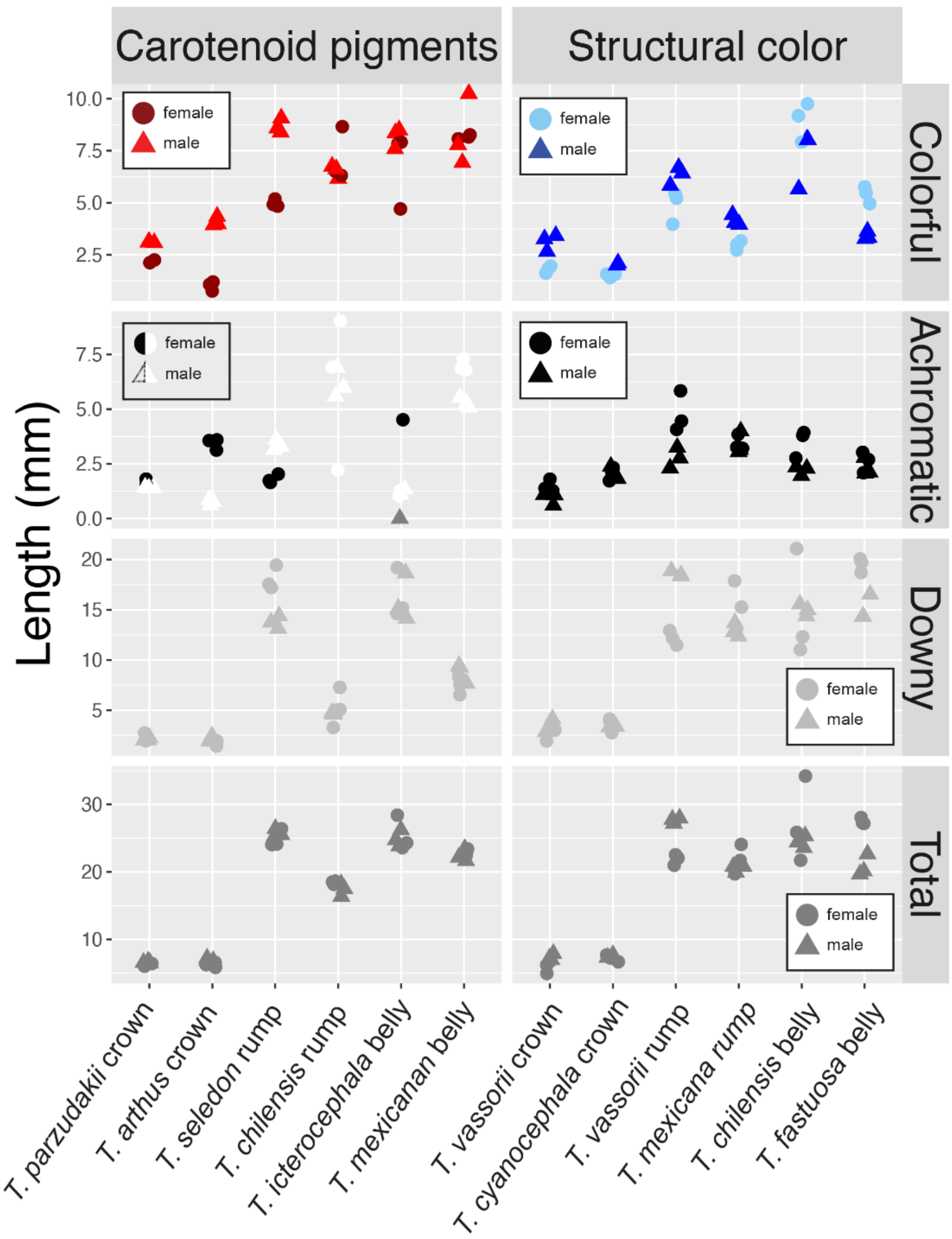


**Figure S12**. **Lengths of colorful, achromatic, and downy regions of each *Tangara* feather in our dataset by coloration mechanism and sex.** Each of the three feather replicates for each patch and sex was measured independently by two authors. Achromatic regions are colored according to their actual color (white versus black), including the three carotenoid patches where females had black rather than white achromatic layers. The triangle outlined in a dashed line indicates the measurement from a male *T. icterocephala* feather that lacked an achromatic region.

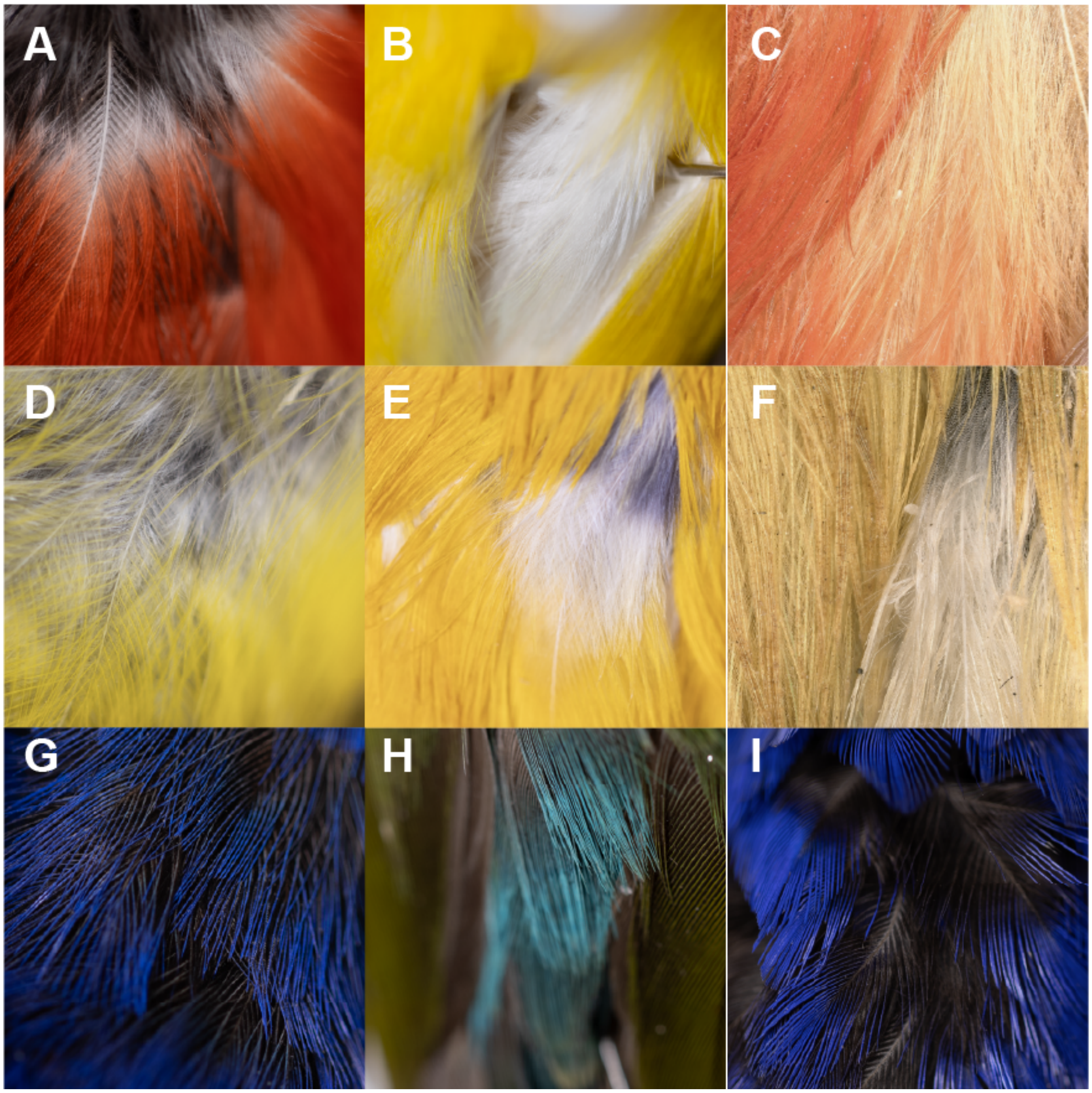


**Figure S13. White achromatic layers are paired with carotenoid-pigmented plumage (A – F) and black achromatic layers are paired with structurally colored plumage (G – I) across passerines.** Images shown above are for species examined (table S5) but not shown in Fig. 5. (A) *Peltops blainvillii* rump, LACM 73381. (B) *Chloebia gouldiae* belly, LACM 766. (C) *Malurus melanocephalus* back, LACM 33592. (D) *Devioeca papuana* belly, LACM 107110. (E) *Lamprotornis regius* belly, LACM 17122. (F) *Dacnis lineata* belly, LACM 85956. (G) *Malurus lamberti* crown, LACM 33610. (H) *Chloebia gouldiae* rump, LACM 766. (I) *Niltava sundara* crown, LACM 74522.

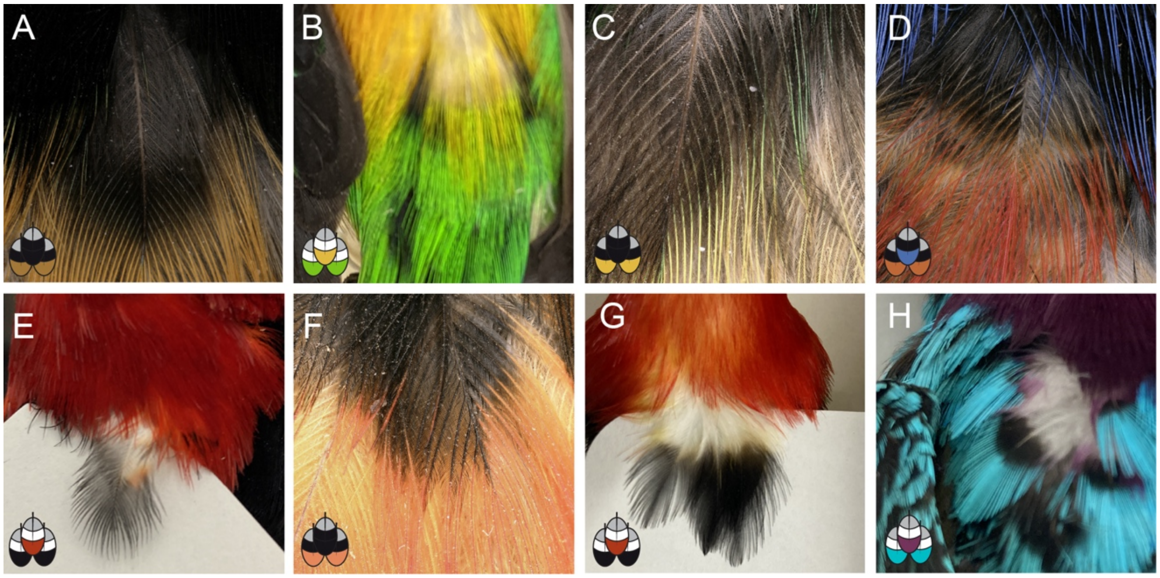


**Figure S14**. **Feather achromatic regions change in color at the junctions of contrasting patches in *Tangara* and other passerines.** Carotenoid-pigmented feathers (A, C, D, F) have black achromatic regions when melanin-pigmented (A, C, F) or structurally colored (D) feathers are layered on top, while structurally colored feathers (B, H) and melanin-pigmented feathers (E) have white achromatic regions when carotenoid-pigmented feathers are layered on top. (A) Female *Tangara fastuosa* rump (carotenoid) x back (melanin). (B) Male *Tangara seledon* upper tail coverts (structural color and carotenoid) x rump (carotenoids). (C) Male *Tangara seledon* rump (carotenoid) x back (melanin). (D) Male *Dacnis berlepschi* belly (carotenoid) x breast (structural color). (E) Male *Ramphocelus nigrogularis* belly (melanin) x breast (carotenoid). (F) Female *Anisognathus igniventris* belly (carotenoid) x breast (melanin). (G) Male *Pipra filicauda* back (melanin) x crown (carotenoid). (H) Male *Cotinga cayana* breast (structural color) x throat (carotenoid). Specimens are from the collections of the Natural History Museum of Los Angeles County (LACM), the Academy of Natural Sciences of Drexel University (ANSP), and the Princeton Museum of Zoology (PMZ). (A) LACM 20582; (B) ANSP 774095; (C) LACM 53514; (D) LACM 37455; (E) ANSP 164412; (F) LACM 42050; (G) ANSP 185641; (H) PMZ 9972.

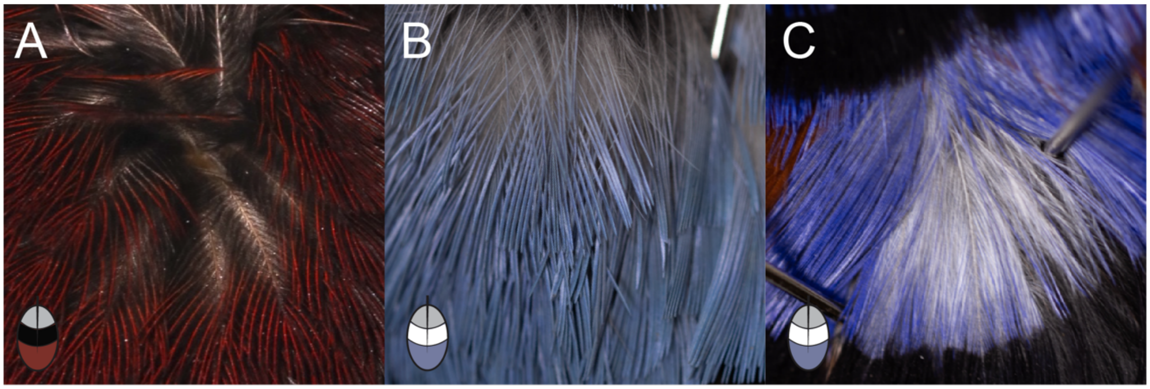


**Figure S15. Black achromatic layer paired with carotenoid plumage (A) and white achromatic layers paired with structurally colored plumage (B-C).** (A) Male silver-beaked tanager (*Ramphocelus carbo*) crown; (B) Male superb fairywren (*Malurus cyaneus*) back, (C) Male variegated fairywren (*Malurus lamberti*) mantle. These pairings result in dark carotenoid-pigmented plumage in (A) and light, unsaturated structurally colored plumage in (B-C), likely produced in conjunction with specific features of the barbs and barbules in the colorful region of the feather. (A) LACM 73266; (B) LACM 33601; (C) LACM 33610.

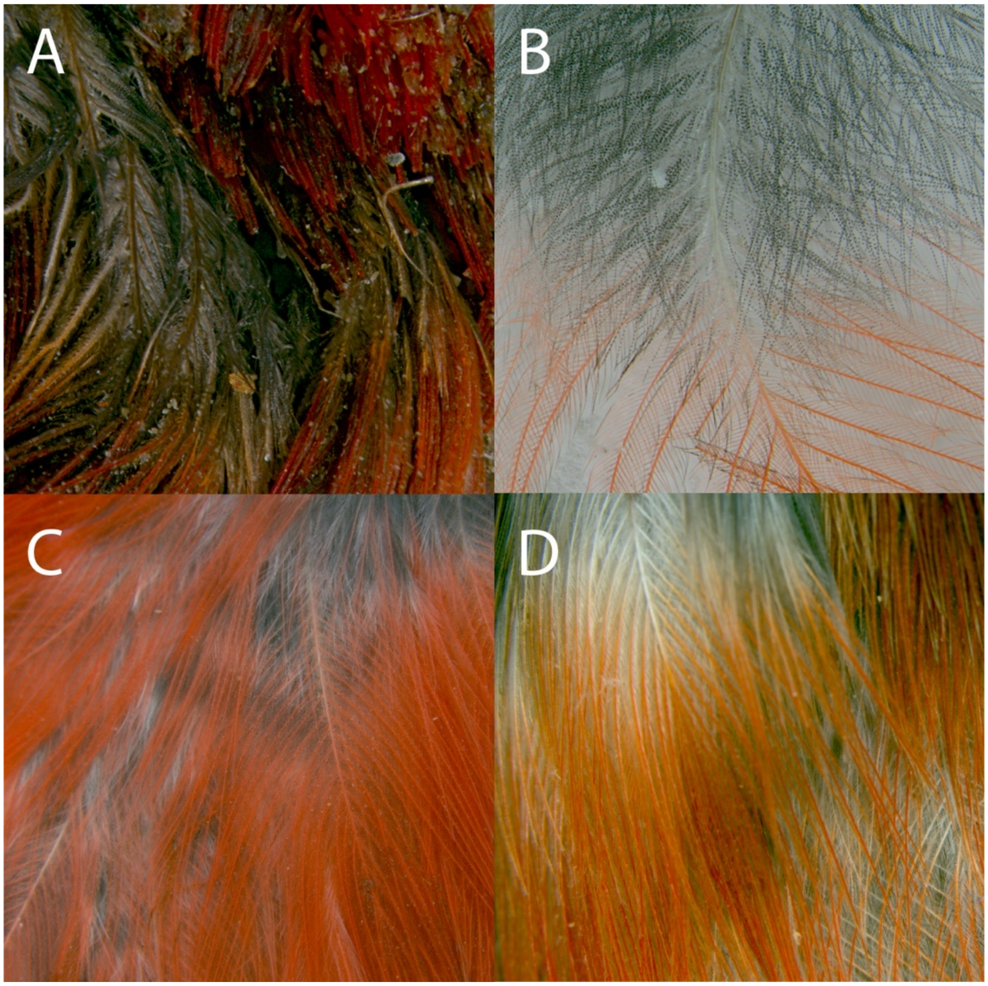


**Figure S16**. **Carotenoid-pigmented feathers lacking achromatic regions.** (A) shows the crown of a male house finch (*Haemorhous mexicanus*) and (B) shows the crown of a male northern cardinal (*Cardinalis cardinalis*). Small white achromatic regions are present in the house finch rump (C) and northern cardinal rump (D). (A,C) PMZ 17002; (B, D) PMZ 16469.

**Table S1. *Tangara* patch and feather sampling scheme for this project.** We picked six carotenoid-pigmented patches and six structurally colored patches from nine *Tangara* species and sampled three feathers per patch for females and males from specimens at the Natural History Museum of Los Angeles (database acronym LACM) and the Academy of Natural Sciences of Drexel University (database acronym ANSP). We also sampled several feathers from the junctions of carotenoid patches (*T. chilensis* rump and *T. cyanocephala* neck) with melanin (*T. chilensis* back) or structural patches (*T. cyanocephala* crown) to visualize changes in the achromatic region color of these feathers (see fig. S14 for further documentation of this phenomenon in *Tangara* and other passerines). 'Mechanism' column refers to the coloration mechanism in the colorful region of the feather.

| Mechanism | Species | Sex | Patch | Voucher | Feathers |
|---|---|---|---|---|---|
| carotenoid | *Tangara icterocephala* | f | belly | LACM 4840 | 3 |
| carotenoid | *Tangara icterocephala* | m | belly | LACM 4842 | 3 |
| carotenoid | *Tangara mexicana* | f | belly | LACM 44617 | 3 |
| carotenoid | *Tangara mexicana* | m | belly | LACM 44616 | 3 |
| carotenoid | *Tangara arthus* | f | crown | LACM 29397 | 3 |
| carotenoid | *Tangara arthus* | m | crown | LACM 29396 | 3 |
| carotenoid | *Tangara parzudakii* | f | crown | LACM 36838 | 3 |
| carotenoid | *Tangara parzudakii* | m | crown | LACM 36839 | 3 |
| carotenoid | *Tangara chilensis* | f | rump | ANSP 116143 | 3 |
| carotenoid | *Tangara chilensis* | m | rump | LACM 85951 | 3 |
| carotenoid | *Tangara chilensis* | m | rump-back junction | LACM 85951 | 3 |
| carotenoid | *Tangara seledon* | f | rump | LACM 53517 | 3 |
| carotenoid | *Tangara seledon* | m | rump | LACM 52514 | 3 |
| carotenoid | *Tangara cyanocephala* | m | neck-crown junction | LACM 27643 | 3 |
| structural | *Tangara chilensis* | f | belly | ANSP 116143 | 3 |
| structural | *Tangara chilensis* | m | belly | LACM 85951 | 3 |
| structural | *Tangara fastuosa* | f | belly | LACM 20582 | 3 |
| structural | *Tangara fastuosa* | m | belly | LACM 60421 | 3 |
| structural | *Tangara cyanocephala* | f | crown | LACM 27645 | 3 |
| structural | *Tangara cyanocephala* | m | crown | LACM 27643 | 3 |
| structural | *Tangara vassorii* | f | crown | LACM 29451 | 3 |
| structural | *Tangara vassorii* | m | crown | LACM 29452 | 3 |
| structural | *Tangara mexicana* | f | rump | LACM 44617 | 3 |
| structural | *Tangara mexicana* | m | rump | LACM 44616 | 3 |
| structural | *Tangara vassorii* | f | rump | LACM 29451 | 3 |
| structural | *Tangara vassorii* | m | rump | LACM 29452 | 3 |

**Table S2. Lightness, saturation, and hue for colorful feather regions on white and black achromatic backgrounds measured with multispectral photography.** For each image, we calculated lightness, saturation, and hue from a cylindrical HSL color space as described in the main text. ΔLightness, saturation, and hue values are measured as the value on white minus the value on black. Mean and SD values for ΔHue are calculated based on the absolute value of ΔHue.

| **Mechanism** | **Species** | **Sex** | **Patch** | **Lightness (%) - white** | **Lightness (%) - black** | **ΔLightness** | **Saturation (%) - white** | **Saturation (% )- black** | **ΔSaturation** | **Hue (°) - white** | **Hue (°) - black** | **ΔHue (°)** |
|---|---|---|---|---|---|---|---|---|---|---|---|---|
| **Multispectral photography (MP)** | | | | | | | | | | | | |
| car | *T. icterocephala* | f | belly | 51.9 | 7.7 | 44.2 | 18.2 | 30.4 | -12.2 | 45.4 | 46.0 | -0.6 |
| car | *T. icterocephala* | m | belly | 51.9 | 7.2 | 44.7 | 17.4 | 27.3 | -9.9 | 47.8 | 47.7 | 0.2 |
| car | *T. mexicana* | f | belly | 59.6 | 12.8 | 46.8 | 18.9 | 21.3 | -2.5 | 51.7 | 45.8 | 5.9 |
| car | *T. mexicana* | m | belly | 58.0 | 13.1 | 44.9 | 19.4 | 23.8 | -4.5 | 50.3 | 45.1 | 5.1 |
| car | *T. arthus* | f | crown | 46.5 | 7.7 | 38.8 | 20.5 | 39.2 | -18.6 | 33.9 | 32.4 | 1.4 |
| car | *T. arthus* | m | crown | 41.6 | 9.5 | 32.1 | 50.3 | 53.4 | -3.2 | 33.1 | 30.6 | 2.5 |
| car | *T. parzudakii* | f | crown | 43.9 | 7.9 | 36.0 | 40.8 | 51.6 | -10.8 | 33.2 | 30.5 | 2.7 |
| car | *T. parzudakii* | m | crown | 46.1 | 9.6 | 36.5 | 46.9 | 47.8 | -1.0 | 31.7 | 28.8 | 2.9 |
| car | *T. chilensis* | f | rump | 40.9 | 8.1 | 32.8 | 52.5 | 62.4 | -9.9 | 14.0 | 11.5 | 2.5 |
| car | *T. chilensis* | m | rump | 33.5 | 7.6 | 25.9 | 59.3 | 61.9 | -2.6 | 13.9 | 10.8 | 3.1 |
| car | *T. seledon* | f | rump | 53.3 | 8.6 | 44.7 | 37.4 | 44.9 | -7.5 | 37.4 | 34.2 | 3.2 |
| car | *T. seledon* | m | rump | 50.0 | 8.6 | 41.4 | 35.2 | 45.1 | -9.9 | 36.3 | 33.1 | 3.2 |
| | | | **Mean** | **48.1** | **9.0** | **39.1** | **34.7** | **42.4** | **-7.7** | **35.7** | **33.0** | **2.8** |
| | | | **SD** | **7.5** | **2.0** | **6.5** | **15.4** | **14.2** | **5.2** | **12.4** | **12.4** | **1.6** |
| str | *T. chilensis* | f | belly | 54.6 | 22.9 | 31.7 | 32.6 | 49.1 | -16.5 | 201.8 | 200.5 | 1.3 |
| str | *T. chilensis* | m | belly | 46.8 | 17.0 | 29.8 | 29.2 | 51.6 | -22.3 | 203.1 | 203.4 | -0.3 |
| str | *T. fastuosa* | f | belly | 40.9 | 7.5 | 33.4 | 5.5 | 23.6 | -18.1 | 216.6 | 223.7 | -7.1 |
| str | *T. fastuosa* | m | belly | 38.3 | 8.7 | 29.6 | 7.1 | 27.3 | -20.2 | 229.8 | 225.4 | 4.4 |
| str | *T. cyanocephala* | f | crown | 34.3 | 13.9 | 20.4 | 28.3 | 51.4 | -23.1 | 221.7 | 219.8 | 1.8 |
| str | *T. cyanocephala* | m | crown | 33.9 | 13.4 | 20.5 | 25.9 | 50.3 | -24.4 | 223.8 | 223.2 | 0.6 |
| str | *T. vassorii* | f | crown | 46.1 | 10.1 | 36.1 | 11.1 | 28.0 | -16.9 | 214.2 | 213.4 | 0.8 |
| str | *T. vassorii* | m | crown | 45.6 | 10.8 | 34.8 | 9.8 | 28.2 | -18.5 | 219.8 | 215.5 | 4.3 |
| str | *T. mexicana* | f | rump | 37.6 | 14.6 | 23.0 | 10.6 | 29.9 | -19.3 | 215.8 | 215.6 | 0.2 |
| str | *T. mexicana* | m | rump | 37.4 | 15.1 | 22.3 | 11.7 | 30.3 | -18.6 | 217.5 | 218.0 | -0.5 |
| str | *T. vassorii* | f | rump | 36.6 | 14.9 | 21.7 | 17.0 | 36.7 | -19.6 | 216.8 | 216.1 | 0.7 |
| str | *T. vassorii* | m | rump | 46.4 | 15.1 | 31.3 | 12.4 | 36.6 | -24.2 | 217.6 | 217.3 | 0.3 |
| | | | **Mean** | **41.5** | **13.7** | **27.9** | **16.8** | **36.9** | **-20.1** | **216.5** | **216.0** | **1.9** |
| | | | **SD** | **6.3** | **4.1** | **5.9** | **9.6** | **10.8** | **2.7** | **7.8** | **7.5** | **2.2** |

**Table S3. Brightness, saturation, and hue for carotenoid-pigmented (car) and structurally colored (str) feather regions on white and black achromatic backgrounds measured with microspectrophotometry and hyperspectral imaging.** For each reflectance spectrum, we measured the brightness (mean reflectance across the spectral range, B2 in pavo) and the saturation (($R_{max}$-$R_{min}$)/mean reflectance; S8 in pavo). ΔBrightness, saturation, and hue values are measured as the value on white minus the value on black. Mean and SD values for ΔHue are calculated based on the absolute value of ΔHue.

| Mechanism | Species | Sex | Patch | Brightness (%) - white | Brightness (%) - black | Δ Brightness | Saturation - white | Saturation - black | Δ Saturation | Hue (nm) - white | Hue (nm) - black | Δ Hue |
|---|---|---|---|---|---|---|---|---|---|---|---|---|
| **Microspectrophotometry (MSP)** | | | | | | | | | | | | |
| car | *T. icterocephala* | f | belly | 50.0 | 30.0 | 20.0 | 1.2 | 0.9 | 0.3 | 700.0 | 551.0 | 149.0 |
| car | *T. icterocephala* | m | belly | 45.0 | 32.5 | 12.5 | 0.9 | 0.8 | 0.0 | 542.0 | 551.0 | -9.0 |
| car | *T. mexicana* | f | belly | 51.5 | 34.5 | 17.0 | 0.7 | 0.4 | 0.3 | 700.0 | 700.0 | 0.0 |
| car | *T. mexicana* | m | belly | 49.5 | 36.5 | 13.0 | 0.6 | 0.5 | 0.1 | 700.0 | 603.0 | 97.0 |
| car | *T. arthus* | f | crown | 43.2 | 29.0 | 14.2 | 1.2 | 0.9 | 0.3 | 700.0 | 660.0 | 40.0 |
| car | *T. arthus* | m | crown | 44.4 | 30.9 | 13.6 | 1.3 | 1.1 | 0.2 | 696.0 | 676.0 | 20.0 |
| car | *T. parzudakii* | f | crown | 37.1 | 26.2 | 10.9 | 1.1 | 0.9 | 0.3 | 673.0 | 700.0 | -27.0 |
| car | *T. parzudakii* | m | crown | 33.2 | 24.9 | 8.3 | 1.1 | 0.8 | 0.3 | 700.0 | 700.0 | 0.0 |
| car | *T. chilensis* | f | rump | 40.9 | 24.0 | 16.8 | 1.7 | 0.9 | 0.8 | 685.0 | 700.0 | -15.0 |
| car | *T. chilensis* | m | rump | 35.2 | 22.6 | 12.6 | 1.6 | 0.9 | 0.7 | 691.0 | 300.0 | 391.0 |
| car | *T. seledon* | f | rump | 45.8 | 27.7 | 18.1 | 1.2 | 0.7 | 0.5 | 700.0 | 300.0 | 400.0 |
| car | *T. seledon* | m | rump | 39.8 | 27.7 | 12.0 | 1.1 | 0.7 | 0.5 | 659.0 | 300.0 | 359.0 |
| | | | **Mean** | **43.0** | **28.9** | **14.1** | **1.1** | **0.8** | **0.3** | **678.8** | **561.8** | **125.6** |
| | | | **SD** | **5.9** | **4.2** | **3.3** | **0.3** | **0.2** | **0.2** | **45.0** | **167.0** | **161.6** |
| str | *T. chilensis* | f | belly | 83.3 | 70.8 | 12.6 | 1.0 | 1.1 | -0.2 | 513.0 | 512.0 | 1.0 |
| str | *T. chilensis* | m | belly | 80.7 | 73.1 | 7.6 | 1.1 | 1.2 | -0.1 | 499.0 | 506.0 | -7.0 |
| str | *T. fastuosa* | f | belly | 44.9 | 37.4 | 7.5 | 1.0 | 1.3 | -0.3 | 377.0 | 382.0 | -5.0 |
| str | *T. fastuosa* | m | belly | 45.0 | 38.7 | 6.3 | 0.8 | 1.2 | -0.4 | 391.0 | 385.0 | 6.0 |
| str | *T. cyanocephala* | f | crown | 50.2 | 45.4 | 4.8 | 1.1 | 1.2 | -0.1 | 449.0 | 448.0 | 1.0 |
| str | *T. cyanocephala* | m | crown | 54.5 | 48.4 | 6.2 | 1.0 | 1.3 | -0.3 | 431.0 | 430.0 | 1.0 |
| str | *T. vassorii* | f | crown | 57.3 | 54.2 | 3.0 | 0.7 | 1.0 | -0.2 | 412.0 | 402.0 | 10.0 |
| str | *T. vassorii* | m | crown | 53.1 | 48.5 | 4.6 | 0.5 | 0.9 | -0.4 | 399.0 | 377.0 | 22.0 |
| str | *T. mexicana* | f | rump | 52.0 | 45.1 | 6.9 | 0.8 | 1.0 | -0.2 | 386.0 | 381.0 | 5.0 |
| str | *T. mexicana* | m | rump | 55.1 | 46.5 | 8.6 | 0.9 | 1.1 | -0.2 | 384.0 | 375.0 | 9.0 |
| str | *T. vassorii* | f | rump | 57.7 | 50.6 | 7.1 | 0.9 | 1.0 | -0.1 | 398.0 | 390.0 | 8.0 |
| str | *T. vassorii* | m | rump | 57.4 | 53.1 | 4.4 | 0.9 | 1.0 | -0.1 | 389.0 | 383.0 | 6.0 |
| | | | **Mean** | **57.6** | **51.0** | **6.6** | **0.9** | **1.1** | **-0.2** | **419.0** | **414.3** | **6.8** |
| | | | **SD** | **12.2** | **11.0** | **2.5** | **0.2** | **0.1** | **0.1** | **45.7** | **49.5** | **5.7** |
| **Hyperspectral Imaging (HI)** | | | | | | | | | | | | |
| car | *T. chilensis* | m | rump | 19.4 | 2.2 | 17.2 | 1.9 | 2.4 | -0.5 | 700.0 | 700.0 | 0.0 |
| str | *T. chilensis* | m | belly | 31.9 | 22.1 | 9.8 | 1.0 | 1.8 | -0.8 | 530.0 | 529.0 | 1.0 |

**Table S4. Differences in female and male *Tangara* coloration for the six carotenoid-pigmented patches and six structurally colored patches in this study.** We measured the color of each patch using UV-vis reflectance spectrophotometry of museum specimens. For each reflectance spectrum, we measured the brightness (mean reflectance across the spectral range, B2 in pavo) and the saturation (($R_{max}$-$R_{min}$)/mean reflectance; S8 in pavo). For each patch, we report sexual dichromatism in brightness and saturation as the male value minus the female value (ΔBrightness and ΔSaturation). We also used avian visual modeling to calculate luminance and chromatic contrasts (ΔL and ΔS) between male and female plumage reflectance spectra.

| Mechanism | Species | Patch | ΔBrightness (Male - Female) | ΔSaturation (Male - Female) | Luminance contrasts (ΔL) | Chromatic contrasts (ΔS) |
|---|---|---|---|---|---|---|
| carotenoid | *T. icterocephala* | belly | -2.61 | 0.19 | 0.52 | 3.13 |
| carotenoid | *T. mexicana* | belly | 1.34 | 0.00 | 0.63 | 1.87 |
| carotenoid | *T. arthus* | crown | 4.68 | 0.25 | 4.11 | 8.88 |
| carotenoid | *T. parzudakii* | crown | 4.09 | -0.62 | 5.73 | 18.06 |
| carotenoid | *T. chilensis* | rump | -0.87 | -0.43 | 0.15 | 2.89 |
| carotenoid | *T. seledon* | rump | 9.20 | 0.14 | 3.33 | 2.01 |
| structural | *T. chilensis* | belly | 17.14 | 0.27 | 3.36 | 2.13 |
| structural | *T. fastuosa* | belly | 7.02 | -0.02 | 3.19 | 1.04 |
| structural | *T. cyanocephala* | crown | -0.91 | -0.07 | 1.13 | 0.79 |
| structural | *T. vassorii* | crown | 9.00 | 0.32 | 2.37 | 2.17 |
| structural | *T. mexicana* | rump | 0.59 | 0.06 | 0.23 | 0.76 |
| structural | *T. vassorii* | rump | 9.32 | 0.14 | 2.14 | 1.04 |

**Table S5. Passerine sampling. We surveyed the pairing of visible layers of plumage with hidden achromatic layers of plumage in passerine museum specimens.** We examined lineages with carotenoid-pigmented or non-iridescent structurally colored plumage, as well as lineages with feathers colored by melanin pigments (eumelanin or pheomelanin). Sex designations are female (f), male (m), or undetermined (u). Specimens were from the Natural History Museum of Los Angeles County (LACM), the Academy of Natural Sciences of Drexel University (ANSP), and the Princeton Museum of Zoology (PMZ).

| Family | Genus | Species | Sex | Patch | Patch color | Patch coloration mechanism | Achromatic region color | Voucher |
|---|---|---|---|---|---|---|---|---|
| Artamidae | *Peltops* | *blainvillii* | m | Rump | Red | Carotenoid | White | LACM 73381 |
| Cardinalidae | *Cardinalis* | *cardinalis* | m | Crown | Red | Carotenoid | Absent | PMZ 16469 |
| Cardinalidae | *Cardinalis* | *cardinalis* | m | Rump | Red | Carotenoid | White | PMZ 16469 |
| Cotingidae | *Cotinga* | *amabilis* | m | Throat | Purple | Carotenoid | White | LACM 77881 |
| Cotingidae | *Cotinga* | *amabilis* | m | Rump | Blue | Structural | Black | LACM 77881 |
| Cotingidae | *Cotinga* | *cayana* | m | Breast | Blue | Structural | Black | PMZ 9972 |
| Estrildidae | *Chloebia* | *gouldiae* | u | Belly | Yellow | Carotenoid | White | LACM 766 |
| Estrildidae | *Chloebia* | *gouldiae* | u | Rump | Blue | Structural | Black | LACM 766 |
| Fringillidae | *Haemorhous* | *mexicanus* | m | Crown | Red | Carotenoid | Absent | PMZ 17002 |
| Fringillidae | *Haemorhous* | *mexicanus* | m | Rump | Red | Carotenoid | White | PMZ 17002 |
| Furnariidae | *Dendroma* | *rufa* | f | Belly | Brown | Pheomelanin | Gray | LACM 27280 |
| Icteridae | *Gymnomystax* | *mexicanus* | f | Belly | Yellow | Carotenoid | White | LACM 36074 |
| Maluridae | *Malurus* | *cyaneus* | m | Mantle | Pale blue | Structural | White | LACM 33601 |
| Maluridae | *Malurus* | *lamberti* | m | Mantle | Blue | Structural | White | LACM 33610 |
| Maluridae | *Malurus* | *lamberti* | m | Crown | Blue | Structural | Black | LACM 33610 |
| Maluridae | *Malurus* | *melanocephalus* | m | Back | Red | Carotenoid | White | LACM 33592 |
| Muscicapidae | *Melaenornis* | *chocolatinus* | f | Belly | Buffy white | Pheomelanin | Pale Gray | LACM 60929 |
| Muscicapidae | *Niltava* | *sundara* | m | Crown | Blue | Structural | Black | LACM 74522 |
| Passerellidae | *Zonotrichia* | *leucophrys* | f | Belly | Buffy white | Pheomelanin | Pale Gray | LACM 111863 |
| Petroicidae | *Devioeca* | *papuana* | f | Belly | Yellow | Carotenoid | White | LACM 107110 |
| Pipridae | *Chiroxiphia* | *caudata* | m | Crown | Red | Carotenoid | White | LACM 94018 |
| Pipridae | *Chiroxiphia* | *caudata* | m | Crown | Dark blue | Structural | Gray | LACM 94018 |
| Pipridae | *Lepidothrix* | *coronata* | m | Crown | Blue | Structural | Black | LACM 36522 |
| Pipridae | *Pipra* | *filicauda* | m | Crown | Red | Carotenoid | White | ANSP 185641 |
| Pittidae | *Erythropitta* | *erythrogaster* | u | Belly | Red | Carotenoid | White | LACM 66188 |
| Pittidae | *Erythropitta* | *erythrogaster* | u | Breast | Blue | Structural | Black | LACM 66188 |
| Sturnidae | *Lamprotornis* | *regius* | u | Belly | Yellow | Carotenoid | White | LACM 17122 |
| Thamnophilidae | *Cercomacroides* | *nigrescens* | m | Belly | Dark gray | Eumelanin | Black | LACM 32003 |

## Table S5 (continued)

| **Family** | **Genus** | **Species** | **Sex** | **Patch** | **Patch color** | **Patch coloration mechanism** | **Achromatic region color** | **Voucher** |
|---|---|---|---|---|---|---|---|---|
| Thraupidae | *Anisognathus* | *igniventris* | f | Belly | Red | Carotenoid | White | LACM 42050 |
| Thraupidae | *Dacnis* | *berlepschi* | m | Belly | Red | Carotenoid | White | LACM 37455 |
| Thraupidae | *Dacnis* | *lineata* | m | Belly | Yellow | Carotenoid | White | LACM 85956 |
| Thraupidae | *Ramphocelus* | *carbo* | m | Crown | Dark red | Carotenoid | Black | LACM 73266 |
| Thraupidae | *Ramphocelus* | *nigrogularis* | m | Breast | Red | Carotenoid | White | ANSP 164412 |
| Thraupidae | *Ramphocelus* | *passerinii* | m | Rump | Red | Carotenoid | White | LACM 14648 |

## REFERENCES AND NOTES


1. S. Johnsen, Hidden in plain sight: The ecology and physiology of organismal transparency. *Biol. Bull.* **201**, 301–318 (2001).

2. J. B. Barnett, C. Michalis, H. M. Anderson, B. L. McEwen, J. Yeager, J. N. Pruitt, N. E. Scott-Samuel, I. C. Cuthill, Imperfect transparency and camouflage in glass frogs. *Proc. Natl. Acad. Sci. U.S.A.* **117**, 12885–12890 (2020).

3. D. Gomez, C. Pinna, J. Pairraire, M. Arias, J. Barbut, A. Pomerantz, W. Daney de Marcillac, S. Berthier, N. Patel, C. Andraud, M. Elias, Wing transparency in butterflies and moths: Structural diversity, optical properties, and ecological relevance. *Ecological monographs* **91**, e01475 (2021).

4. D. E. McCoy, T. Feo, T. A. Harvey, R. O. Prum, Structural absorption by barbule microstructures of super black bird of paradise feathers. *Nat. Commun.* **9**, 1 (2018).

5. D. E. McCoy, V. E. McCoy, N. K. Mandsberg, A. V. Shneidman, J. Aizenberg, R. O. Prum, D. Haig, Structurally assisted super black in colourful peacock spiders. *Proc. Biol. Sci.* **286**, 20190589 (2019).

6. A. L. Davis, H. F. Nijhout, S. Johnsen, Diverse nanostructures underlie thin ultra-black scales in butterflies. *Nat. Commun.* **11**, 1294 (2020).

7. B. D. Wilts, K. Michielsen, H. De Raedt, D. G. Stavenga, Sparkling feather reflections of a bird-of-paradise explained by finite-difference time-domain modeling. *Proc. Natl. Acad. Sci. U.S.A.* **111**, 4363–4368 (2014).

8. B.-K. Hsiung, R. H. Siddique, D. G. Stavenga, J. C. Otto, M. C. Allen, Y. Liu, Y.-F. Lu, D. D. Deheyn, M. D. Shawkey, T. A. Blackledge, Rainbow peacock spiders inspire miniature super-iridescent optics. *Nat. Commun.* **8**, 2278 (2017).

9. B. D. Wilts, V. Saranathan, A literal elytral rainbow: Tunable structural colors using single diamond biophotonic crystals in *Pachyrrhynchus congestus* weevils. *Small* **14**, e1802328 (2018).

10. R. Price-Waldman, M. C. Stoddard, Avian coloration genetics: Recent advances and emerging questions. *J. Hered.* **112**, 395–416 (2021).

11. I. C. Cuthill, W. L. Allen, K. Arbuckle, B. Caspers, G. Chaplin, M. E. Hauber, G. E. Hill, N. G. Jablonski, C. D. Jiggins, A. Kelber, J. Mappes, J. Marshall, R. Merrill, D. Osorio, R. Prum, N. W. Roberts, A. Roulin, H. M. Rowland, T. N. Sherratt, J. Skelhorn, M. P. Speed, M. Stevens, M. C. Stoddard, D. Stuart-Fox, L. Talas, E. Tibbetts, T. Caro, The biology of color. *Science* **357**, eaan0221 (2017).

12. T. Caro, M. C. Stoddard, D. Stuart-Fox, Animal coloration research: Why it matters. *Philos. Trans. R. Soc. Lond. B Biol. Sci.* **372**, 20160333 (2017).

13. N. M. Cruz, R. M. White, Lessons on transparency from the glassfrog. *Science* **378**, 1272–1273 (2022).

14. G. E. Hill, Plumage coloration is a sexually selected indicator of male quality. *Nature* **350**, 337–339 (1991).

15. W. D. Hamilton, M. Zuk, Heritable true fitness and bright birds: A role for parasites? *Science* **218**, 384–387 (1982).

16. P. O. Dunn, J. K. Armenta, L. A. Whittingham, Natural and sexual selection act on different axes of variation in avian plumage color. *Sci. Adv.* **1**, e1400155 (2015).

17. R. S. Terrill, A. J. Shultz, Feather function and the evolution of birds. *Biol. Rev.* **98**, 540–566 (2023).

18. M. C. Stoddard, R. O. Prum, How colorful are birds? Evolution of the avian plumage color gamut. *Behav. Ecol.* **22**, 1042–1052 (2011).

19. K. J. McGraw, “Mechanisms of carotenoid-based coloration” in *Bird Coloration, Volume 1: Mechanisms and Measurements*, G. E. Hill, K. J. McGraw, Eds. (Harvard Univ. Press, 2006), pp. 177–242.

20. R. O. Prum, "Anatomy, physics, and evolution of avian structural colors" in *Bird Coloration, Volume 1: Mechanisms and Measurements*, G. E. Hill, K. J. McGraw, Eds. (Harvard Univ. Press, 2006), pp. 295–353.

21. V. Saranathan, J. D. Forster, H. Noh, S.-F. Liew, S. G. J. Mochrie, H. Cao, E. R. Dufresne, R. O. Prum, Structure and optical function of amorphous photonic nanostructures from avian feather barbs: A comparative small angle x-ray scattering (SAXS) analysis of 230 bird species. *J. R. Soc. Interface* **9**, 2563–2580 (2012).

22. K. Delhey, M. Valcu, J. Dale, B. Kempenaers, The evolution of carotenoid-based plumage colours in passerine birds. *J. Anim. Ecol.* **92**, 66–77 (2023).

23. M. D. Shawkey, L. D'Alba, Interactions between colour-producing mechanisms and their effects on the integumentary colour palette. *Philos. Trans. R. Soc. Lond. B Biol. Sci.* **372**, 20160536 (2017).

24. A. M. Lucas, P. Stettenheim, "Growth and color of feathers" in *Avian Anatomy Integument, Volume 2* (Avian Anatomy Project, Poultry Research Branch, Animal Science Research Division, Agricultural Research Service, U.S. Department of Agriculture, 1972), pp. 341–419.

25. A. Wetmore, The number of contour feathers in passeriform and related birds. *The Auk* **53**, 159–169 (1936).

26. R. O. Prum, Development and evolutionary origin of feathers. *J. Exp. Zool.* **285**, 291–306 (1999).

27. M. D. Shawkey, G. E. Hill, Carotenoids need structural colours to shine. *Biol. Lett.* **1**, 121–124 (2005).

28. S. Andersson, M. Prager, "Quantifying colors" in *Bird Coloration, Volume. 1: Mechanisms and Measurement*, G. E. Hill, K. J. McGraw, Eds. (Harvard Univ. Press, 2006), pp. 41–89.

29. S. Johnsen, *The Optics of Life: A Biologist's Guide to Light in Nature* (Princeton Univ. Press, 2012).

30. Q. O. N. Kay, H. S. Daoud, C. H. Stirton, Pigment distribution, light reflection and cell structure in petals. *Bot. J. Linn. Soc.* **83**, 57–83 (1981).

31. N. I. Morehouse, P. Vukusic, R. Rutowski, Pterin pigment granules are responsible for both broadband light scattering and wavelength selective absorption in the wing scales of pierid butterflies. *Proc. Biol. Sci.* **274**, 359–366 (2006).

32. D. G. Stavenga, C. J. van der Kooi, Coloration of the Chilean Bellflower, N*olana paradoxa*, interpreted with a scattering and absorbing layer stack model. *Planta* **243**, 171–181 (2016).

33. C. J. van der Kooi, J. T. M. Elzenga, M. Staal, D. G. Stavenga, How to colour a flower: On the optical principles of flower coloration. *Proc. Biol. Sci.* **283**, 20160429 (2016).

34. M. D. Shawkey, G. E. Hill, K. J. McGraw, W. R. Hood, K. Huggins, An experimental test of the contributions and condition dependence of microstructure and carotenoids in yellow plumage coloration. *Proc. Biol. Sci.* **273**, 2985–2991 (2006).

35. M. D. Shawkey, G. E. Hill, Significance of a basal melanin layer to production of non-iridescent structural plumage color: Evidence from an amelanotic Steller's jay (*Cyanocitta stelleri*). *J. Exp. Biol.* **209**, 1245–1250 (2006).

36. S. Yoshioka, S. Kinoshita, Structural or pigmentary? Origin of the distinctive white stripe on the blue wing of a *Morpho* butterfly. *Proc. R. Soc. B* **273**, 129–134 (2006).

37. D. G. Stavenga, H. L. Leertouwer, B. D. Wilts, Coloration principles of nymphaline butterflies – thin films, melanin, ommochromes and wing scale stacking. *J. Exp. Biol.* **217**, 2171–2180 (2014).

38. B. Igic, L. D'Alba, M. D. Shawkey, Fifty shades of white: How white feather brightness differs among species. *Sci. Nat.* **105**, 18 (2018).

39. D. E. McCoy, R. O. Prum, Convergent evolution of super black plumage near bright color in 15 bird families. *J. Exp. Biol.* **222**, jeb208140 (2019).

40. L. Saks, K. McGraw, P. Hõrak, How feather colour reflects its carotenoid content. *Funct. Ecol.* **17**, 555–561 (2003).

41. M. D. Shawkey, A. M. Estes, L. Siefferman, G. E. Hill, The anatomical basis of sexual dichromatism in non-iridescent ultraviolet-blue structural coloration of feathers. *Biol. J. Linn. Soc.* **84**, 259–271 (2005).

42. D. E. McCoy, A. J. Shultz, J. E. Dall, J. A. Dionne, S. Johnsen, The carotenoid redshift: Physical basis and implications for visual signaling. *Ecol. Evol.* **13**, e10408 (2023).

43. G. F. Grether, G. R. Kolluru, K. Nersissian, Individual colour patches as multicomponent signals. *Biol. Rev.* **79**, 583–610 (2004).

44. B. Casalía, E. Vilacoba, P. D. Lavinia, P. L. Tubaro, A. S. Barreira, UV sensitive vision in cardinals and tanagers is ubiquitous. *Emu - Austral Ornithol.* **120**, 355–359 (2021).

45. A. Ödeen, O. Håstad, The phylogenetic distribution of ultraviolet sensitivity in birds. *BMC Evol. Biol.* **13**, 36 (2013).

46. M. C. Stoddard, R. O. Prum, Evolution of avian plumage color in a tetrahedral color space: A phylogenetic analysis of New World buntings. *Am. Nat.* **171**, 755–776 (2008).

47. C. D. Jones, D. Osorio, Discrimination of oriented visual textures by poultry chicks. *Vision Res.* **44**, 83–89 (2004).

48. M. L. Isler, P. R. Isler, *The Tanagers* (Smithsonian International Press, 1999).

49. M. Vorobyev, D. Osorio, Receptor noise as a determinant of colour thresholds. *Proc. R. Soc. Lond. B Biol. Sci.* **265**, 351–358 (1998).

50. A. Siddiqi, T. W. Cronin, E. R. Loew, M. Vorobyev, K. Summers, Interspecific and intraspecific views of color signals in the strawberry poison frog *Dendrobates pumilio*. *J. Exp. Biol.* **207**, 2471–2485 (2004).

51. C. N. Spottiswoode, M. Stevens, Visual modeling shows that avian host parents use multiple visual cues in rejecting parasitic eggs. *Proc. Natl. Acad. Sci. U.S.A.* **107**, 8672–8676 (2010).

52. J. Dale, C. J. Dey, K. Delhey, B. Kempenaers, M. Valcu, The effects of life history and sexual selection on male and female plumage colouration. *Nature* **527**, 367–370 (2015).

53. C. R. Cooney, Z. K. Varley, L. O. Nouri, C. J. A. Moody, M. D. Jardine, G. H. Thomas, Sexual selection predicts the rate and direction of colour divergence in a large avian radiation. *Nat. Commun.* **10**, 1773 (2019).

54. C. J. Schmitt, S. V. Edwards, Passerine birds. *Curr. Biol.* **32**, R1149–R1154 (2022).

55. S. M. Doucet, D. J. Mennill, G. E. Hill, The evolution of signal design in manakin plumage ornaments. *Am. Nat.* **169**, S62–S80 (2007).

56. C. Alfonso, B. C. Jones, B. J. Vernasco, I. T. Moore, Integrative studies of sexual selection in manakins, a clade of charismatic tropical birds. *Integr. Comp. Biol.* **61**, 1267–1280 (2021).

57. J. S. Berv, R. O. Prum, A comprehensive multilocus phylogeny of the Neotropical cotingas (Cotingidae, Aves) with a comparative evolutionary analysis of breeding system and plumage dimorphism and a revised phylogenetic classification. *Mol. Phylogenet. Evol.* **81**, 120–136 (2014).

58. R. J. Weaver, E. S. A. Santos, A. M. Tucker, A. E. Wilson, G. E. Hill, Carotenoid metabolism strengthens the link between feather coloration and individual quality. *Nat. Commun.* **9**, 73 (2018).

59. G. E. Hill, "Female mate choice for ornamental coloration" in *Bird Coloration Vol 2: Function and Evolution*, G. E. Hill, K. J. McGraw, Eds. (Harvard Univ. Press, 2006), pp. 137–200.

60. S. Rogalla, M. D. Shawkey, L. D'Alba, Thermal effects of plumage coloration. *Ibis* **164**, 933–948 (2022).

61. D. Stuart-Fox, E. Newton, R. A. Mulder, L. D’Alba, M. D. Shawkey, B. Igic, The microstructure of white feathers predicts their visible and near-infrared reflectance properties. *PLOS ONE* **13**, e0199129 (2018).

62. A. Bernabe, K. J. Burns, Blue-and-black tanager (*Tangara vassorii*), version 1.0., *Birds of the World* (2020). https://doi.org/10.2173/bow.babtan1.01.

63. S. Barve, V. Ramesh, T. M. Dotterer, C. J. Dove, Elevation and body size drive convergent variation in thermo-insulative feather structure of Himalayan birds. *Ecography* **44**, 680–689 (2021).

64. M. P. J. Nicolaï, M. D. Shawkey, S. Porchetta, R. Claus, L. D’Alba, Exposure to UV radiance predicts repeated evolution of concealed black skin in birds. *Nat. Commun.* **11**, 2414 (2020).

65. M. P. J. Nicolaï, R. Vanisterbecq, M. D. Shawkey, L. D’Alba, Back in black: Melanin-rich skin colour associated with increased net diversification rates in birds. *Biol. Lett.* **19**, 20230304 (2023).

66. C. R. Cooney, Y. He, Z. K. Varley, L. O. Nouri, C. J. A. Moody, M. D. Jardine, A. Liker, T. Székely, G. H. Thomas, Latitudinal gradients in avian colourfulness. *Nat. Ecol. Evol.* **6**, 622–629 (2022).

67. A. V. Badyaev, Altitudinal variation in sexual dimorphism: A new pattern and alternative hypotheses. *Behav. Ecol.* **8**, 675–690 (1997).

68. J. K. Hubbard, Z. W. D. Williard, Spectra of feather samples are impacted by the substrate color against which they are measured. *Wilson J. Ornithol.* **135**, 1–9 (2023).

69. R. O. Prum, R. Torres, Structural colouration of avian skin: Convergent evolution of coherently scattering dermal collagen arrays. *J. Exp. Biol.* **206**, 2409–2429 (2003).

70. L. M. Mäthger, R. T. Hanlon, Malleable skin coloration in cephalopods: Selective reflectance, transmission and absorbance of light by chromatophores and iridophores. *Cell Tissue Res.* **329**, 179–186 (2007).

71. T. L. Williams, S. L. Senft, J. Yeo, F. J. Martín-Martínez, A. M. Kuzirian, C. A. Martin, C. W. DiBona, C.-T. Chen, S. R. Dinneen, H. T. Nguyen, C. M. Gomes, J. J. C. Rosenthal, M. D. MacManes, F. Chu, M. J. Buehler, R. T. Hanlon, L. F. Deravi, Dynamic pigmentary and structural coloration within cephalopod chromatophore organs. *Nat. Commun.* **10**, 1004 (2019).

72. E. Twomey, M. Kain, M. Claeys, K. Summers, S. Castroviejo-Fisher, I. Van Bocxlaer, Mechanisms for color convergence in a mimetic radiation of poison frogs. *Am. Nat.* **195**, E132–E149 (2020).

73. R. O. Prum, R. H. Torres, Structural colouration of mammalian skin: Convergent evolution of coherently scattering dermal collagen arrays. *J. Exp. Biol.* **207**, 2157–2172 (2004).

74. T. Sai, B. D. Wilts, A. Sicher, U. Steiner, F. Scheffold, E. R. Dufresne, When black and white make green: The surprising interplay of structure and pigments. *Chimia* **73**, 47–50 (2019).

75. M. Iwata, M. Teshima, T. Seki, S. Yoshioka, Y. Takeoka, Bio-inspired bright structurally colored colloidal amorphous array enhanced by controlling thickness and black background. *Adv. Mater.* **29**, 1605050 (2017).

76. M. Xiao, Z. Hu, Z. Wang, Y. Li, A. D. Tormo, N. Le Thomas, B. Wang, N. C. Gianneschi, M. D. Shawkey, A. Dhinojwala, Bioinspired bright noniridescent photonic melanin supraballs. *Sci. Adv.* **3**, e1701151 (2017).

77. R. Maia, H. Gruson, J. A. Endler, T. E. White, pavo 2: New tools for the spectral and spatial analysis of colour in r. *Methods Ecol. Evol.* **10**, 1097–1107 (2019).

78. R Core Team, R: A language and environment for statistical computing, version 4.4.0, R Foundation for Statistical Computing (2024); https://R-project.org.

79. J. Troscianko, M. Stevens, Image calibration and analysis toolbox—A free software suite for objectively measuring reflectance, colour and pattern. *Methods Ecol. Evol.* **6**, 1320–1331 (2015).

80. C. A. Schneider, W. S. Rasband, K. W. Eliceiri, NIH Image to ImageJ: 25 years of image analysis. *Nat. Methods* **9**, 671–675 (2012).

81. S. Barthelmé, D. Tschumperlé, imager: An R package for image processing based on CImg. *J. Open Source Softw.* **4**, 1012 (2019).

82. B. G. Hogan, M. C. Stoddard, Hyperspectral imaging in animal coloration research: A user-friendly pipeline for image generation, analysis, and integration with 3D modeling. *PLOS Biol.* **22**, e3002867 (2024).

83. C. H. Oliveros, D. J. Field, D. T. Ksepka, F. K. Barker, A. Aleixo, M. J. Andersen, P. Alström, B. W. Benz, E. L. Braun, M. J. Braun, G. A. Bravo, R. T. Brumfield, R. T. Chesser, S. Claramunt, J. Cracraft, A. M. Cuervo, E. P. Derryberry, T. C. Glenn, M. G. Harvey, P. A. Hosner, L. Joseph, R. T. Kimball, A. L. Mack, C. M. Miskelly, A. T. Peterson, M. B. Robbins, F. H. Sheldon, L. F. Silveira, B. T. Smith, N. D. White, R. G. Moyle, B. C. Faircloth, Earth history and the passerine superradiation. *Proc. Natl. Acad. Sci. U.S.A.* **116**, 201813206 (2019).

84. S. U. Linville, R. Breitwisch, A. J. Schilling, Plumage brightness as an indicator of parental care in northern cardinals. *Anim. Behav.* **55**, 119–127 (1998).

85. C. J. Dove, S. L. Koch, Microscopy of feathers: A practical guide for forensic feather identification. *The Microscope* **59**, 51–71 (2011).

86. R. Bleiweiss, Phenotypic integration expressed by carotenoid-bearing plumages of tanager-finches (Thraupini, Emberizinae) across the avian visible spectrum. *Biol. J. Linn. Soc.* **93**, 89–109 (2008).

87. M. B. Toomey, K. L. Ronald, Avian color expression and perception: Is there a carotenoid link? *J. Exp. Biol.* **224**, jeb203844 (2021).

88. O. Lind, M. J. Henze, A. Kelber, D. Osorio, Coevolution of coloration and colour vision? *Philos. Trans. R. Soc. B Biol. Lond. Sci.* **372**, 20160338 (2017).

89. D.-J. Jeon, S. Ji, E. Lee, J. Kang, J. Kim, L. D'Alba, M. Manceau, M. D. Shawkey, J.-S. Yeo, How keratin cortex thickness affects iridescent feather colours. *R. Soc. Open Sci.* **10**, 220786 (2023).

90. M. D. Shawkey, A. M. Estes, L. M. Siefferman, G. E. Hill, Nanostructure predicts intraspecific variation in ultraviolet–blue plumage colour. *Proc. R. Soc. Lond. B Biol. Sci.* **270**, 1455–1460 (2003).

91. M. Fan, L. D'alba, M. D. Shawkey, A. Peters, K. Delhey, Multiple components of feather microstructure contribute to structural plumage colour diversity in fairy-wrens. *Biol. J. Linn. Soc.* **128**, 550–568 (2019).

92. D. E. McCoy, A. V. Shneidman, A. L. Davis, J. Aizenberg, Finite-difference time-domain (FDTD) optical simulations: A primer for the life sciences and bio-inspired engineering. *Micron* **151**, 103160 (2021).
